# Single-cell 3D genome structure reveals distinct human pluripotent states

**DOI:** 10.1186/s13059-024-03268-w

**Published:** 2024-05-13

**Authors:** Niannian Li, Kairang Jin, Bin Liu, Mingzhu Yang, PanPan Shi, Dai Heng, Jichang Wang, Lin Liu

**Affiliations:** 1 State Key Laboratory of Medicinal Chemical Biology, Nankai University, 94 Weijin Road, Tianjin, 300071 China; 2 Weifang People’s Hospital, Shandong, 261041 China; 3 Department of Cell Biology and Genetics, College of Life Sciences, Nankai University, 94 Weijin Road, Tianjin, 300071 China; 4 Key Laboratory for Stem Cells and Tissue Engineering (Sun Yat-Sen University), Ministry of Education, Guangzhou, 510080 China; 5 Department of Histology and Embryology, Zhongshan School of Medicine, Sun Yat-Sen University, Guangzhou, 510080 China; 6 TEDA Institute of Biological Sciences and Biotechnology, Nankai University, 23 Hongda Street, TEDA, Tianjin, 300457 China

**Keywords:** Human embryonic stem cells, Pluripotency, Naive, Primed, Genome structure, Chromatin accessibility

## Abstract

**Background:**

Pluripotent states of embryonic stem cells (ESCs) with distinct transcriptional profiles affect ESC differentiative capacity and therapeutic potential. Although single-cell RNA sequencing has revealed additional subpopulations and specific features of naive and primed human pluripotent stem cells (hPSCs), the underlying mechanisms that regulate their specific transcription and that control their pluripotent states remain elusive.

**Results:**

By single-cell analysis of high-resolution, three-dimensional (3D) genomic structure, we herein demonstrate that remodeling of genomic structure is highly associated with the pluripotent states of human ESCs (hESCs). The naive pluripotent state is featured with specialized 3D genomic structures and clear chromatin compartmentalization that is distinct from the primed state. The naive pluripotent state is achieved by remodeling the active euchromatin compartment and reducing chromatin interactions at the nuclear center. This unique genomic organization is linked to enhanced chromatin accessibility on enhancers and elevated expression levels of naive pluripotent genes localized to this region. In contradistinction, the primed state exhibits intermingled genomic organization. Moreover, active euchromatin and primed pluripotent genes are distributed at the nuclear periphery, while repressive heterochromatin is densely concentrated at the nuclear center, reducing chromatin accessibility and the transcription of naive genes.

**Conclusions:**

Our data provide insights into the chromatin structure of ESCs in their naive and primed states, and we identify specific patterns of modifications in transcription and chromatin structure that might explain the genes that are differentially expressed between naive and primed hESCs. Thus, the inversion or relocation of heterochromatin to euchromatin via compartmentalization is related to the regulation of chromatin accessibility, thereby defining pluripotent states and cellular identity.

**Supplementary Information:**

The online version contains supplementary material available at 10.1186/s13059-024-03268-w.

## Background

Although human pluripotent stem cells (hPSCs) and notably embryonic stem cells (ESCs) hold great promise in the regeneration of tissues, conventional hESCs are essentially classified as primed PSCs, like mouse epiblast stem cells (mEpiSCs) that exhibit lower differentiative capability [1–4]—limiting their potentially broad applicability. Significant progress has enabled the conversion of primed hESCs to the naive state of pluripotency, resembling well-characterized naive mouse ESCs (mESCs) [5–11]. Intriguingly, naive hPSCs can generate blastocyst-like structures in vitro under effective three-dimensional (3D) culture conditions [12]. Naive hPSCs express higher levels of specific pluripotent genes such as *OCT4*, *NANOG, STELLA*/*DPPA3,* and *DPPA5*—as well as the specific endogenous retrovirus *HERVH*—whereas primed hPSCs particularly express *ZIC2, OTX2*, and *B3GAT1* [7, 8, 11, 13–17]. While single-cell RNA-sequencing (scRNA-seq) has revealed additional subpopulations and specific features of naive and primed hPSCs [18, 19], the mechanisms that regulate the specific transcription and control of these states have remained elusive. By taking advantage of a single-cell, diploid chromatin conformation-capture method termed Dip-C that reveals sufficiently high-resolution genomic structures [20], we herein demonstrated that the naive pluripotent state was regulated by active euchromatin compartmentalization and fewer chromatin interactions in the interior of the nucleus.

## Results

### Molecular features of pluripotent states as revealed by RNA-seq

**Fig. 1:**
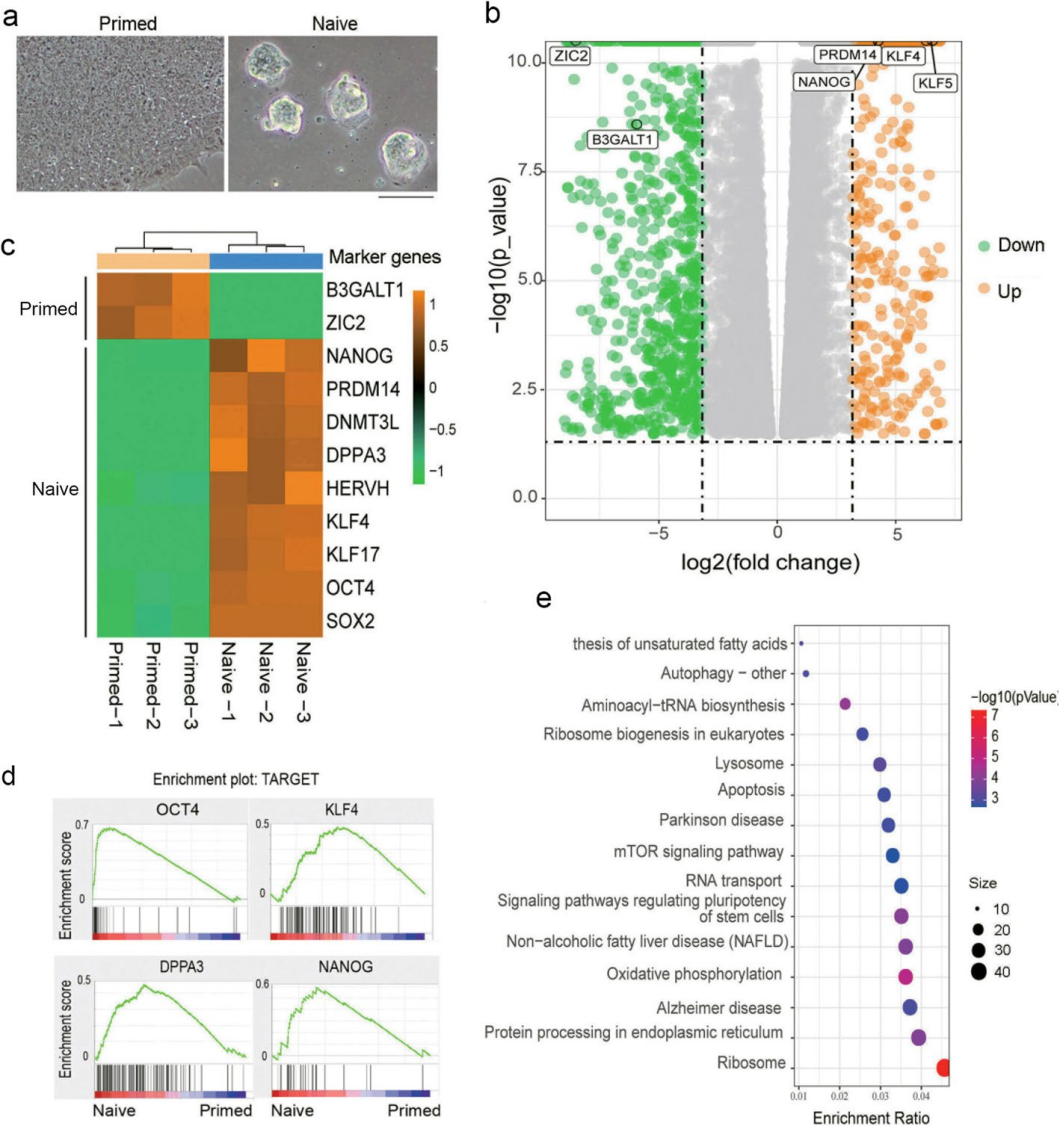
Distinct transcriptional expression profiles of extended naive and primed states of hESCs. **a** Morphology of extended naive and primed hESCs originating from H9 cell lines (scale bar, 100 μm). **b** RNA-seq analysis of differential gene expression between naive and primed hESCs. Red color indicates upregulated genes in naive hESCs; green designates upregulated genes in primed hESCs (representative genes are shown); gray denotes unchanged. **c** Heatmap showing the relative expression of representative marker genes for naive and primed hESCs. **d** GSEA of the target genes of pluripotency-related transcription factors in naive and primed hESCs. **e** KEGG-enrichment analysis of naive and primed hESCs based on our RNA-seq data

The naive pluripotent state is achieved by conversion of conventional (primed) hESCs in a novel culture condition, and the novel naive hESCs exhibit developmental pluripotency by robust human–mouse interspecies chimerism. Morphologically, naive hESCs exhibited small dome-shaped 3D colonies, whereas primed hESCs showed 2D flat, large colonies (Fig. 1a). By exploiting RNA-seq analysis, we observed that the transcriptome of the naive state differed from that of the primed state and expressed naive pluripotent genes at higher levels (Fig. 1b). Quantitative real-time PCR provided validation of marker genes that are used to reflect naive pluripotency, as *NANOG*, *OCT4*, *KLF4*, *DPPA3*, and *HERVH* were expressed at much higher levels in the naive state relative to the primed state, whereas *ZIC2* and *B3GALT1*/*B3GAT1* [21] were expressed at higher levels in the primed state (Fig. 1C and Additional file 1: Fig. S1a, b). Western-blot analysis showed that naive hESCs expressed OCT4 and NANOG at higher levels than primed hESCs (Additional file 1: Fig. S1c). These data were consistent with recent studies showing that naive hPSCs expressed NANOG and OCT4 at markedly higher levels than conventional hESCs [16, 22]. Moreover, target genes regulated by pluripotency transcription factors were significantly enriched in naive hESCs (Fig. 1d). By analysis of the RNA-seq data for significant enrichment of KEGG signaling pathways, highly expressed genes in the naive state were enriched in cellular pluripotency-related, oxidative phosphorylation, ribosomal signaling, and protein synthesis-related pathways (Fig. 1e, and Additional file 1: Fig. S1d, e). Additionally, naive and primed hESCs differed in their activation of signaling pathways related to cell-to-cell connection and transduction (Additional file 1: Fig. S1f). Thus, pluripotent hESCs in their naive and primed states exhibited their own unique gene expression patterns.

### High chromatin intermingling index in the primed state relative to the naive state

**Fig. 2:**
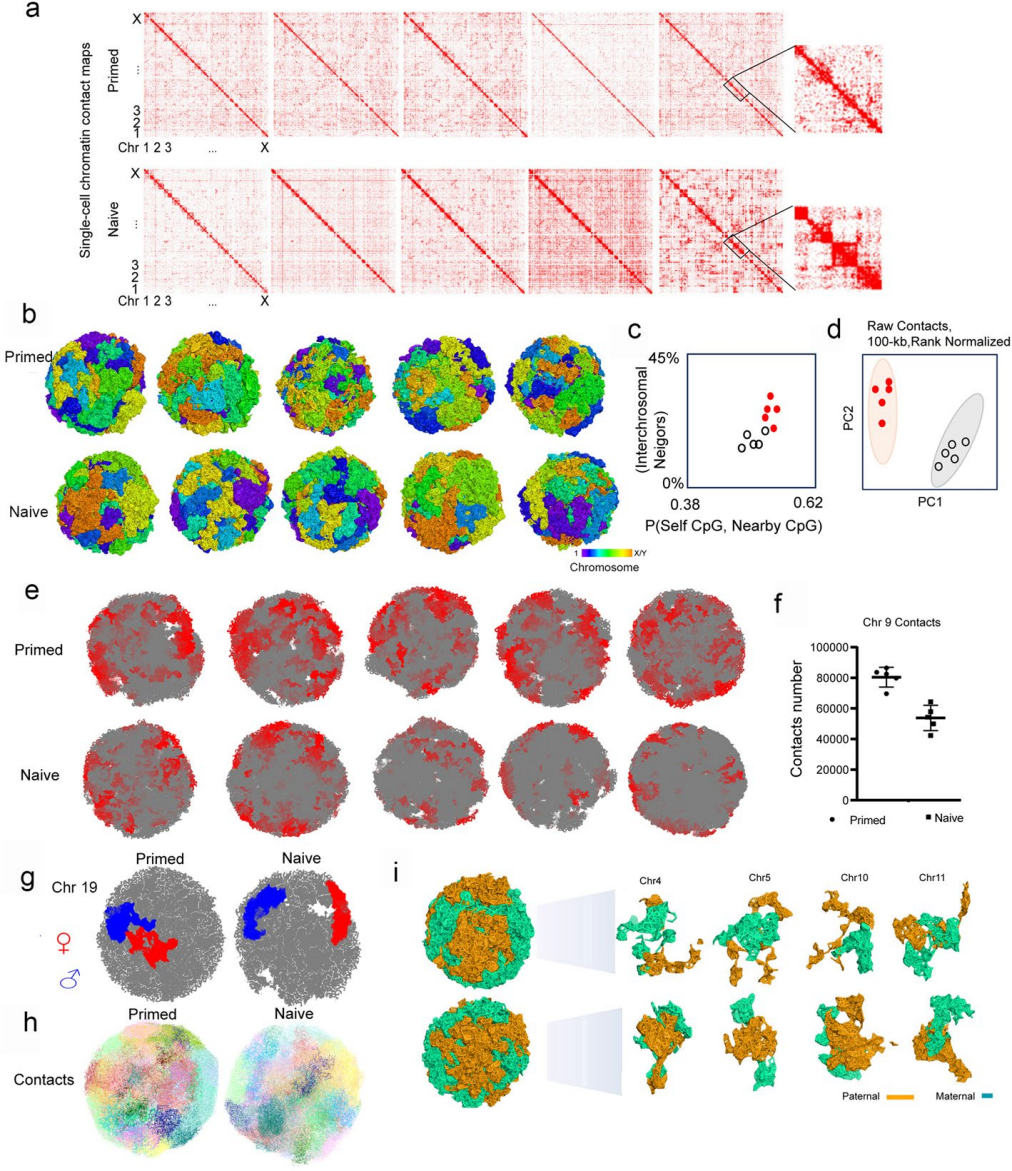
Genomic structure and chromosomal organization of naive and primed hESCs as revealed by single-cell Dip-C. **a** Representative single-cell chromatin contact maps after haplotype imputation showing a scattered distribution of chromatin indicative of more chromatin interactions in the primed state but greater localized chromatin distribution suggestive of fewer chromatin interactions in the naive state (five individual cells are shown). **b** 3D genomic structure of primed and naive states of hESCs that reveal greater intermingling of chromosomes in the primed state but with a more-localized regional distribution of chromosomes in the naive state. Each particle represents 20 kb of chromatin or a radius of ~ 100 nm (five individual cells are shown: the various colored bars indicate chromosome nos. 1, 2, 3.., X/Y; and higher magnification and resolution can be found in Fig. 2f. **c** Quantification of chromosomal intermingling (vertical axis: the average fraction of nearby particles that were not from the same chromosome) and chromatin compartmentalization (horizontal axis: Spearman’s correlation between each particle’s own CpG frequency and the average of nearby particles). **d** The same two clusters can also be distinguished by unsupervised clustering via PCA of single-cell chromatin compartments, without the need for bulk data. The two alleles of each locus were treated as two different loci. **e** Example cross-sections of two cell types, colored by chromosome. **f** The contacts in chromosome 9 of two groups of five cells. **g** The position of genetic information on chromosome 9 in the nucleus from both the paternal and maternal parents. **h** Example cross-sections of two cell types, colored by the multi-chromosome intermingling index. **i** Structural differences in homologous chromosomes from the parents between naive and primed hESCs (chromosomes 4, 5, 10, and 11 are shown; and other chromosomes can be found in Fig. S3A)

To understand how the specific transcription of these two pluripotent states of hESCs is regulated at the chromatin or genomic organizational levels, we assessed genomic organization and analyzed chromatin spatial interactions using single-cell chromatin conformation capture and haplotype imputation by Dip-C [20] (this method is outlined and the results of the library construction are detailed in Additional file 1: Fig. S2a–e). The 3D information on chromatin structure is encoded in the linear genome through proximity ligation of chromatin fragments (as in 3C and Hi-C) and provides novel insights into chromatin spatial organization in individual cells, avoiding the potential masking of differences in chromatin interactions in each cell within a heterogeneous population. After the 2D heatmap was obtained, the 3D chromatin structure was simulated (Fig. 2a, b) based on a previously described method [20] (and also in the “ Methods” section). DNA contacts in the naive state exhibited regular regional localization, but DNA contacts in the primed state showed a more dispersed distribution (Fig. 2a, b, c, d, and Additional file 1: Fig. S2f). Furthermore, from the perspective of overall chromatin structure, chromatin intermingling was higher in the primed state relative to the naive state (Fig. 2b, c). This chromatin intermingling indicates an interaction between chromatins, and more interaction may represent a more-complex regulation related to the differentiation of cells [20]. Notably, chromosomes indicated by various colors were more intermingled in the nuclei of the primed state cells, but specific regional preference for each chromosome was evident in the naive state (Fig. 2b, i and Additional file 1: Fig. S2f). A mixed-chromosome distribution likely implied greater inter-chromosomal interactions, consistent with the higher intermingling noted in primed hESCs. Localized chromosomal organization denotes fewer inter-chromosomal interactions in the naive state (Fig. 2e, f, g, h). For example, chromosome 9 possessed the highest number of genes and the highest intermingling between chromatins, indicating a significant difference between the naive and primed states (Fig. 2 f, g).

We simulated 3D chromatin structures of the 23 pairs of chromosomes in naive and primed cells, and using the Dip-C method, we were able to show for the first time ever the 3D chromatin structure of the diploid chromosomes of pluripotent stem cells. Maternal and paternal chromosomes in naive cells showed a relatively compartmentalized distribution, while chromosomal distribution in primed cells exhibited higher intermingling and more disorganization than in naive cells (Fig. 2e). We also observed another peculiar phenomenon in which the morphology of the maternal and paternal X chromosomes differed in primed cells. The maternal and paternal X chromosomes were not uniform, as the maternal X chromosome was in a more relaxed conformation, and the paternal X chromosome appeared shrunken and spherical (Additional file 1: Fig. S3a), which may have contributed to the activated maternal X chromosome but not to the inactivated paternal X chromosome in primed cells. In contrast, naive cells manifested a relatively uniform distributional state of the two X chromosomes (Additional file 1: Fig. S3a).

### Divergent distributions of the X chromosome in the genomic structure of naive and primed hESCs

**Fig. 3:**
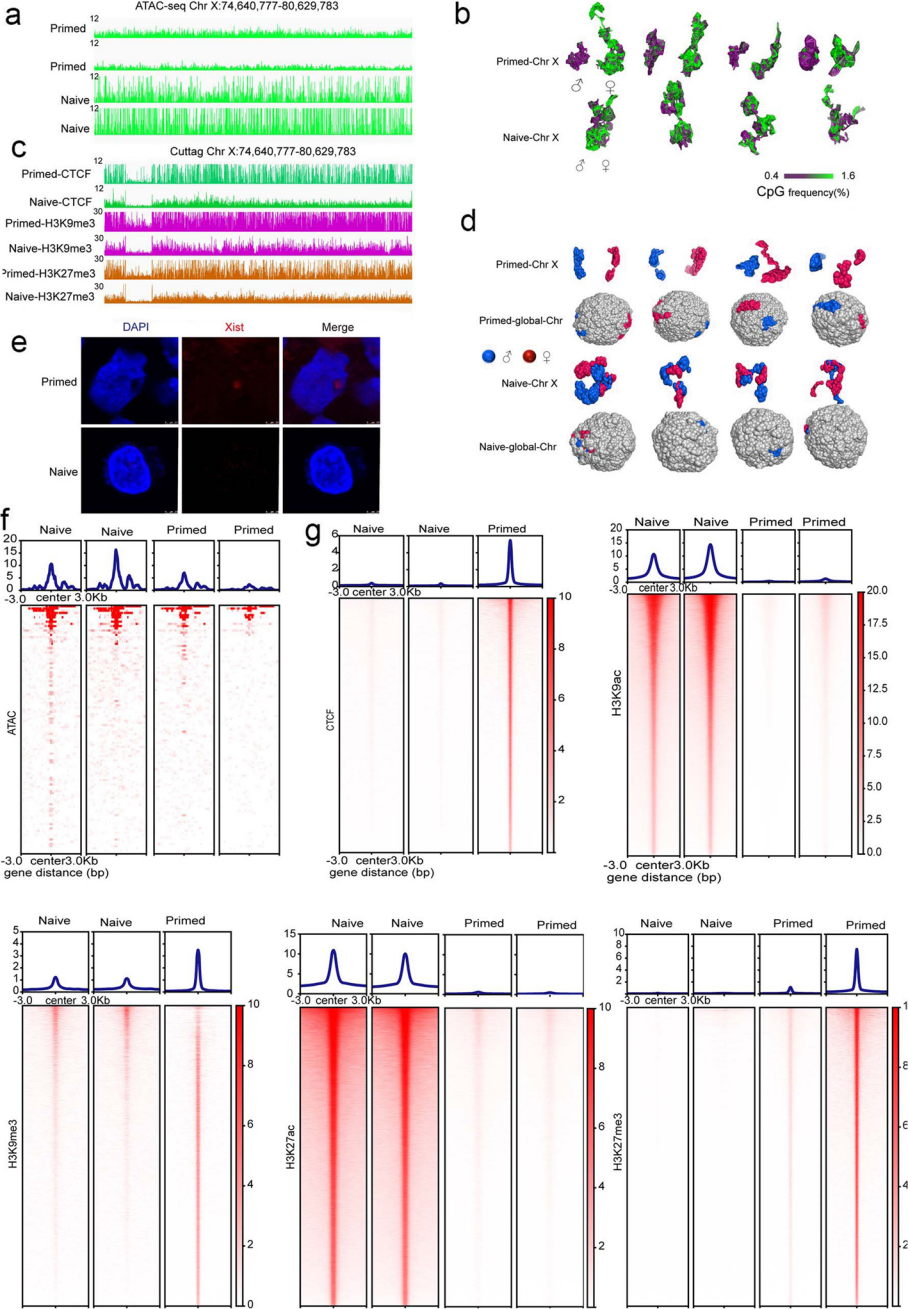
3D structures and features of the maternal and paternal X chromosomes in naive and primed hESCs. **a** ATAC-seq signal tracks for representative genomic regions in the X chromosome; y-axes = reads per million (rpm). **b** 3D structures of four single cells in naive and primed states showing compartmentalization of euchromatin (green) and heterochromatin (purple), as visualized using CpG frequency as a proxy. **c** Representative H3K9me3, H3K27me3, and CTCF landscapes across chrX:74,640,777–80,629,783 of the genome, as generated by CUT&Tag in naive and primed hESCs. **d** Spatial position and structure of the parental X chromosome in the simulated 3D structures of all chromosomes (four individual cells are shown). **e** Representative images of Xist RNA-FISH in primed and naive hESCs (nuclei were stained with DAPI [blue]; scale bar, 5 μm). **f** ATAC-seq signal tracks for the cell as a whole; y-axes = reads per million (rpm). **g** Representative H3K9me3, H3K27me3, and CTCF landscapes of the genome, as generated by CUT&Tag in naive and primed hESCs

To reveal the activation state of the X chromosomes, we analyzed the chromatin accessibility of the two X chromosomes using ATAC-seq in naive and primed PSCs and demonstrated that naive PSCs consistently exhibited greater chromatin accessibility than did primed PSCs (Fig. 3a, f). We also determined the frequency of CpG sites in order to analyze the openness of the chromatin and ascertained that the euchromatin regions were mostly CpG-rich, unlike heterochromatin that was devoid of CpG dinucleotides [20]. Comparison analysis of the frequency of CpGs in paternal and maternal X chromosomes showed a higher frequency of CpG sites on the maternal X chromosome, suggestive of an active state, but a lower frequency of CpGs on the paternal X chromosome, indicative of an inactivated state in primed PSCs (Fig. 3b). Naive PSCs, in contrast, showed a higher frequency of CpGs in both maternal and paternal X chromosomes, suggesting that naive PSCs acquired more active X chromosomes compared to primed PSCs. Furthermore, when we analyzed the histone modifications of X-chromosome regions, we noted that primed PSCs captured more repressive histone modifications, as determined by sequencing information and evidenced by a higher enrichment of CTCF, H3K9me3, and H3K27me3 in the X chromosome in contrast to naive PSCs (Fig. 3c, g). This is likely one of the major mechanisms that explains the differential activities of X chromosomes in the two cell types.

We also simulated the position of the X chromosomes in primed and naive cells based on a previously described methodology [20]. The shrunken paternal X chromosome was embedded within the cell surface, while the activated maternal X chromosome interludes from the nucleus in primed cells. However, two activated X chromosomes were interspersed in the nucleus in a relatively uniform manner in naive cells (Fig. 3d and Additional file 1: Fig. S3c, g). In naive PSCs, two X chromosomes were intertwined and formed a relatively uniform entity, not only in morphology but also in the frequency of CpG sites, indicating that the two X chromosomes possessed similarly elevated activity. By Xist RNA-FISH, Xist was undetectable in the naive cells, consistent with the absence of an inactive X chromosome, while the X chromosome inactivation signal from Xist was identified at the edge of the nucleus in primed cells (Fig. 3e). These results corresponded to those of our Dip-C analysis (Fig. 3d and Additional file 1: Fig. S3c).

### Compartmentalized A–B displacement of naive state versus primed state

**Fig. 4:**
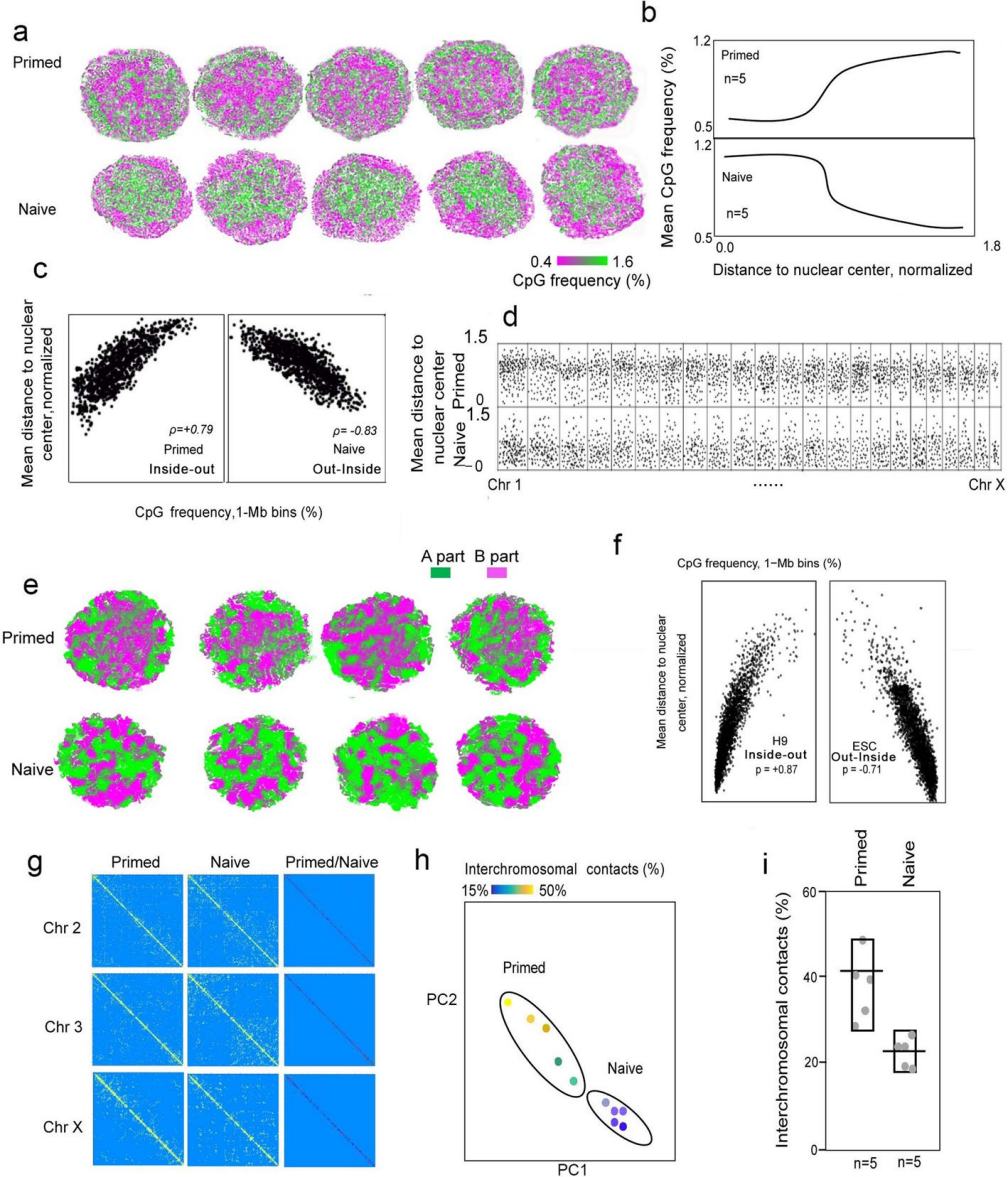
Distinct interchromosomal contacts in naive and primed hESCs. **a** Serial cross-sections of a single cell showing compartmentalization of euchromatin (green) and heterochromatin (magenta), as visualized using CpG frequency as a proxy. Note: all five representative cells yielded a consistent pattern of chromatin compartmentalization in the primed and naive states, with each particle representing 20 kb of chromatin (~ 60 nm in radius). Adjacent serial sections are separated by 7.5 particle radii (~ 450 nm). **b** Radial distribution of CpGs is defined as the mean CpG frequency in each concentric spherical shell, averaged over all single cells of the same cell type. Radial distances (to the nuclear center of mass) in each cell were normalized by their mean. **c** Genome-wide correlation between radial positioning was defined as the normalized distance to the nuclear center of mass and the CpG frequency of consecutive 1-Mb bins. **d** Radial positioning along the genome. Radial preferences across the genome, as measured by average distances to the nuclear center of mass. **e** Lieberman–Aiden’s method was applied to distinguish A/B compartments. **f** Genome-wide correlation between radial positioning was defined as the normalized distance to the nuclear center of mass and the CpG frequency of consecutive 1-Mb bins in bulk-cell Hi-C data of naive and primed hESCs.** g** 2D heatmap of the chromosomal contacts and distributions of naive and primed hESCs. Primed/naive designates that primed reflects specific contacts compared to naive. **h**, **i** Interchromosomal contact map and percentage of interchromosomal contacts of the naive state compared with the primed state. Chromosomal intermingling was quantified as the percentage of interchromosomal contacts that were increased in primed hESCs

An elevated frequency of CpG islands in the DNA was found in the active transcriptional area that represented the euchromatin [20], suggesting an augmented chromatin openness in the naive state compared to the primed state. Remarkably, the euchromatin of the naive state was concentrated in the interior of the nucleus, while the euchromatin was more concentrated at the nuclear periphery, with the central region enriched with heterochromatin, in the primed state (Fig. 4a). Importantly, all individual cells that we analyzed exhibited a similar pattern of chromatin structure and organization for each state (Fig. 4a and Additional file 1: Fig. S3b), implying a generalized feature of chromatin structures in both naive and primed hESCs. We analyzed the relationship between the CpG frequency at the chromatin sites and the distance to the nuclear center and discerned that the higher CpG frequency was found in the primed state, farther away from the central nuclear region. In contrast, the CpG frequency was lower in the naive state, farther away from the nuclear center (Fig. 4b). The distance to the nuclear center in the primed sate was also positively correlated with CpG frequency, which has been designated as an “inside-out” chromatin distribution. In contrast, the distance to the nuclear center in the naive sate was negatively correlated with the CpG frequency, referred to as an “out-inside” chromatin distribution (Fig. 4c). We additionally analyzed bulk cell Hi-C data of naive and primed hESCs to confirm whether some of findings were conservative (Fig. 4f). Compartment A/B as defined by Lieberman-Aiden et al. was also suggested to be mapped to the 3D chromatin architecture so as to compare it with the CpG frequency map (Fig. 4e) [23], and our results revealed a difference from the previous assessment of CpG frequency. There was also a similar trend with respect to primed central aggregation of additional B components. We ascertained that the active euchromatin organization or compartmentalization at the nuclear center of the naive state and the repressive heterochromatic compartmentalization in the central region of the primed sate in hESCs appeared to be active or open chromatin (A) or repressed or closed chromatin (B) compartments, respectively, and that they were distinct from the A-B compartment switch reported previously [23–25].

Hence, chromatin interactions of naive and primed PSCs differed significantly in the A–B compartments. Nevertheless, conversion of primed state to naive state in hESCs appears to be regulated by a dramatic inversion or displacement of heterochromatin to euchromatin compartmentalization that takes place at the nuclear center, a mechanism that is different from the typical A–B compartment switch between the nuclear center and periphery that reflects a model previously found in other cell types (refer to the “ Discussion” below).

### Naive and primed hESCs differ in their contact distributions of DNA interaction sites

**Fig. 5:**
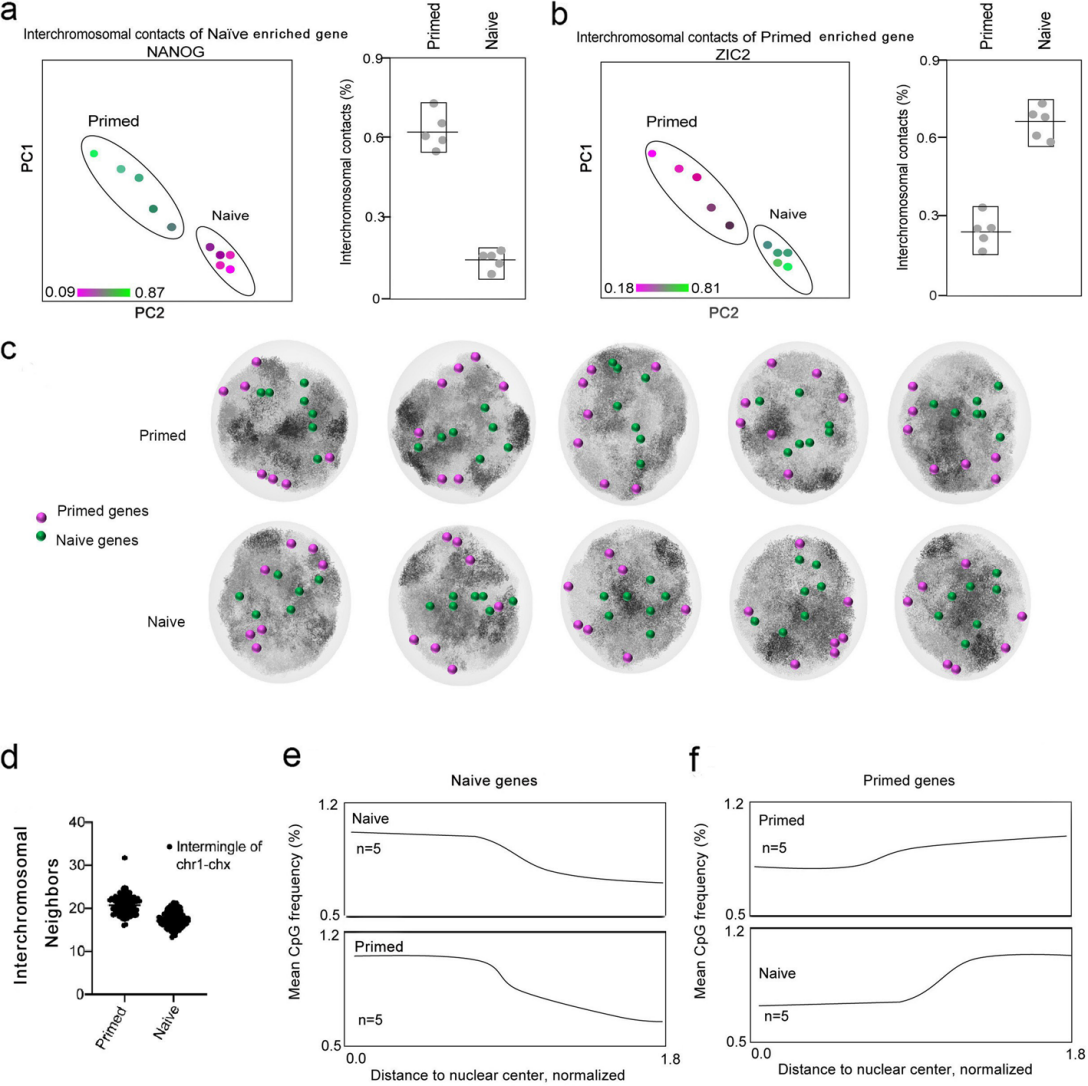
Localization of naive and primed genes vis-a-vis chromatin compartmentalization and their interaction sites. **a** The interaction sites of naive-state enriched genes (e.g., *NANOG*) in the chromatin compartment and their relationships with interchromosomal contacts differed between naive and primed hESCs (example genes were chosen for cell-type-specific chromatin compartment values; not all genes showed such trends).** b** The interaction sites of primed state-enriched genes (e.g., *ZIC2*) in the chromatin compartment and their relationships with interchromosomal contacts differed between naive and primed hESCs (example genes were chosen for cell-state-specific chromatin compartment values; not all genes showed such trends).** c** Distinct distributions of representative 3D genomic structures of naive (toward the nuclear center) and primed genes (peripheralized regions) on the 3D chromatin structure in five single naive cells and five single primed cells. **d** Quantification of chromosomal intermingling (vertical axis: the average fraction of nearby particles that were not from the same chromosome) and chromatin compartmentalization (horizontal axis: Spearman’s correlation between each particle’s own CpG frequency and the average of nearby particles). **e, f** Radial distribution of CpGs averaged over all single cells of the same cell type. Radial distances to the nuclear center of mass in each cell were normalized by their mean

We performed PCA on the chromatin contacts of naive and primed states based on single-cell Dip-C data and demonstrated that the two states were distinctly clustered and separated (Additional file 1: Fig. S3d). According to the statistics governing CpG sites in the chromatin contact locus in a single cell, the distribution of CpGs in primed hESCs was far from the nuclear center and the distribution looser but that of naive hESCs was closer to the nuclear center and more concentrated (Fig. 4d). We uncovered a similar distribution at the chromosomal level by 2D structure (Additional file 1: Fig. S3g). This suggests that active chromatin interactions in the primed state are more inclined to be at the border of the nucleus, and the interaction sites more scattered. Active chromatin interactions in the naive state were more biased toward the central nucleus, and the interaction sites were more locally regulated, confirming the above analysis and showing higher intermingling of chromatin in primed vs. naive states. Based on our observations of the interaction heatmap, the naive state showed notable chromatin interaction regions on autosomes, where a relatively complete topologically associating domain (TAD) structure could be observed; however, the primed state lost its obvious interaction boundaries, and the chromatin interactions tended to be intermingled (Fig. 4g). We counted 2 cell types, with 5 cells for each type, and analyzed the chromatin intermingling of 24 chromosomes within each cell. The intermingling of individual chromosomes in naive and primed cells manifested a relatively unified trend (Fig. 5d), with analyses of the interchromosomal contact numbers in single cells showing that the contacts in the primed state noticeably exceeded those of the naive state (Fig. 4h, i and Fig. 2c, d), consistent with the 3D chromosomal structure (shown in Fig. 2b).

These data further suggest that compared with the regulation by chromatin organization of the naive state, chromatin regulation of the primed state is more complex than previously thought. Differential broad and global genome/chromatin organization could therefore have important implications for regulating the primed and naive states of hESCs.

### Chromatin structure vis-à-vis the expression of pluripotent genes

We next determined how the unique chromatin compartmentalization we observed was linked to the specific expression of pluripotent genes by separately counting the chromatin contacts on and near the loci of highly expressed genes in both naive and primed states. As an example, there appeared to be fewer interchromosomal contacts at the *NANOG* chromatin area in the naive-state cells showing high expression levels of *NANOG* (Fig. 5a). In contrast, fewer interchromosomal contacts were found in the chromatin regions that highly expressed *ZIC2* in primed cells (Fig. 5b). These data showed that these highly expressed genes were located in regions with looser chromatin structure.

We subsequently analyzed the genome-wide gene distribution in association with chromatin organization and consistently showed that the loci of highly expressed genes for the naive state were primarily located in the nuclear core, while those for the primed state were mostly located closer to or along the nuclear edge (Fig. 5c). Highly expressed genes for the two states were also principally localized in or close to their respective active euchromatin regions (Figs. 5c and 4a). For example, representative genes for the primed state (including *ZIC2*, *OTX2*, and *B3GAT1*) were spread away from the nuclear center where euchromatin of the primed state was formed, whereas naive genes (*NANOG*, *KLF4*, and *DPPA3*) were localized at the nuclear center or the approximated center of the euchromatin in the naive state (Additional file 1: Fig. S4a). We further analyzed the relationship between CpG frequency and the position of the genes relative to the nuclear center in the two states and showed that for naive genes in the primed cells, the CpG frequency decreased with increasing distance to the center (Fig. 5e); this indicated that the naive pluripotent genes were localized to the central region of primed hESCs and were enriched with repressive heterochromatin (Fig. 4a). This relationship can partially explain why naive pluripotent genes are suppressed in primed hESCs. For naive pluripotent genes in naive hESCs, the CpG frequency also decreased commensurately with increasing distance to the central region, indicating that the naive pluripotent gene was located in the nuclear core of naive hESCs—i.e., the euchromatin region (Fig. 5e)—corroborating augmented expression of naive pluripotent genes.

The CpG frequency increased with increasing distance to the center (Fig. 5f) in the primed pluripotent genes in primed hESCs, indicating that these genes were located at the nuclear periphery of the primed hESCs where the euchromatin was organized (Fig. 4a). However, for the primed pluripotent genes in naive hESCs, the CpG frequency decreased with increasing distance to the central region, indicating that the primed pluripotent genes were located at the nuclear border of naive hESCs where the heterochromatin was distributed (Fig. 4a); thus, the primed pluripotent genes were not expressed in naive hESCs. In general, the naive pluripotent genes were localized to the central euchromatin region of naive hESCs, and the primed pluripotent genes were mostly concentrated at the nuclear boundary euchromatin region of primed hESCs. This unique compartmentalization explains the differential gene expression of these two pluripotent states.

### Chromatin accessibility facilitated by relaxation of genomic structure

**Fig. 6:**
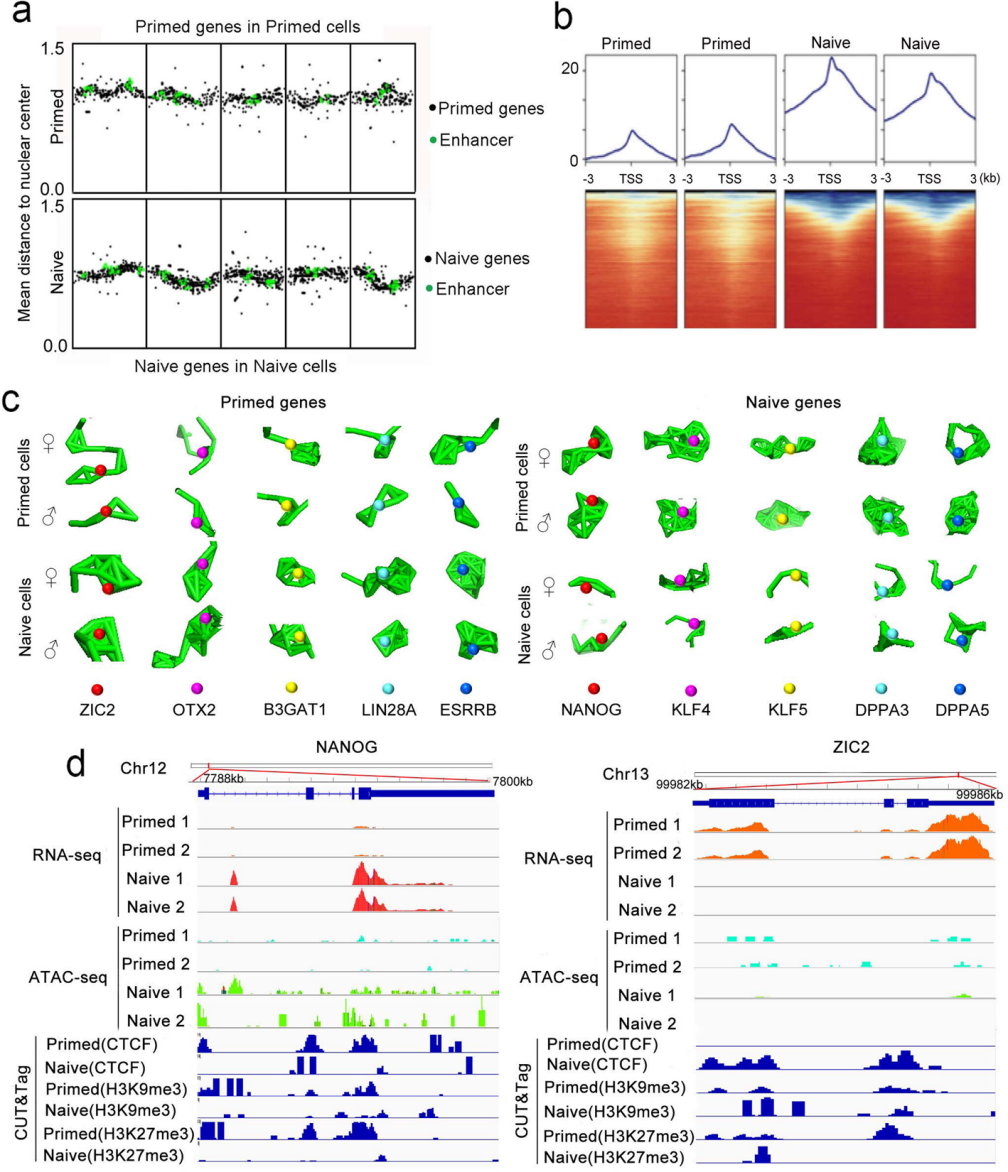
Transcriptional regulation and chromatin accessibility in naive and primed states. **a** Radial positioning along the genome of primed or naive genes and enhancers in naive or primed hESCs.** b** Heatmaps of ATAC-seq signal distribution around the transcriptional start site (TSS) ± 3000 bp of expressed genes and average profiles of the enrichment at the TSS in naive or primed hESCs (two replicates are shown for each state). **c** 3D structural differences between the two alleles of the representative marker-gene loci in primed and naive cells.** d** Joint analysis by ATAC-seq, CUT&Tag, and RNA-seq of the relationships between gene expression and chromatin accessibility in naive (*NANOG*) and primed states (*ZIC2*). Genome browser tracks of RNA-seq, ATAC-seq, and CUT&Tag-seq data of CTCF, H3K9me3, and H3K27me3 at the NANOG and ZIC2 loci in naive and primed hESCs

The enhancer–promoter interaction model constitutes an important component of chromatin structure in the regulation of gene expression. In the euchromatin region, the chromatin arrangement is relatively loose, which provides conditions for the formation and function of the enhancer–promoter interaction. The enhancer region then binds to the transcriptional activators when the chromatin is folded, creating the close spatial distance between the enhancer and the promoter, thus initiating gene expression [26, 27]. To assess whether the expression of pluripotent genes was linked to the enhancer chromatin structure, we analyzed the distribution of the enhancers at the gene loci in primed and naive hESCs. Our data revealed that more enhancers were enriched around the loci of the naive pluripotent genes in naive hESCs, and more enhancers were distributed around the loci of primed pluripotent genes in primed hESCs (Additional file 1: Fig. S4b). Moreover, primed pluripotent genes showed overlap of the loci with the enhancers in primed hESCs, and naive pluripotent genes overlapped at the loci with enhancers in naive hESCs (Fig. 6a, Additional file 1: Fig. S8). These results are consistent with the model in which chromatin is folded to form an enhancer–promoter loop, thus facilitating transcription.

To form an enhancer–promoter loop, a relaxed open chromatin structure is required. Mapping open chromatin using an assay for transposase-accessible chromatin (ATAC-seq) previously revealed distinct chromatin accessibility of naive and primed hPSCs [22]. To explore the consequences of distinct genomic organization of primed and naive states and to discern whether genomic spatial organization facilitated chromatin accessibility, we performed low (cell number) input ATAC-seq on our naive hESCs and compared them with primed hESCs. ATAC-seq detected more open euchromatin structure in naive cells (Fig. 6b), confirming the Dip-C analysis, showing that the naive state possessed a smaller number of chromatin contacts (Fig. 4i). When we simulated the contact morphology of primed and naive pluripotent genes localized in the chromatin based on a previously reported method [20], we demonstrated that chromatin contacts for naive pluripotent genes became relaxed in naive cells but were more closed for primed pluripotent genes in primed cells (Fig. 6c). Combined analysis with ATAC-seq and RNA-seq for the marker genes of two pluripotent states revealed that the *NANOG* gene was highly expressed in naive hESCs and that the chromatin at the gene locus was more open (Fig. 6d). *ZIC2* was also highly expressed in primed hESCs, and the chromatin at the gene locus also depicted more openness. Other naive genes such as *KLF4* and *KLF5* that are highly expressed in naive hESCs also exhibited elevated chromatin openness (Additional file 1: Fig. S4c). Hence, the chromatin structure as it relates to chromatin accessibility and enhancers regulates gene expression in primed and naive hESCs.

### Distribution of histone marks in the two states of hESCs

**Fig. 7:**
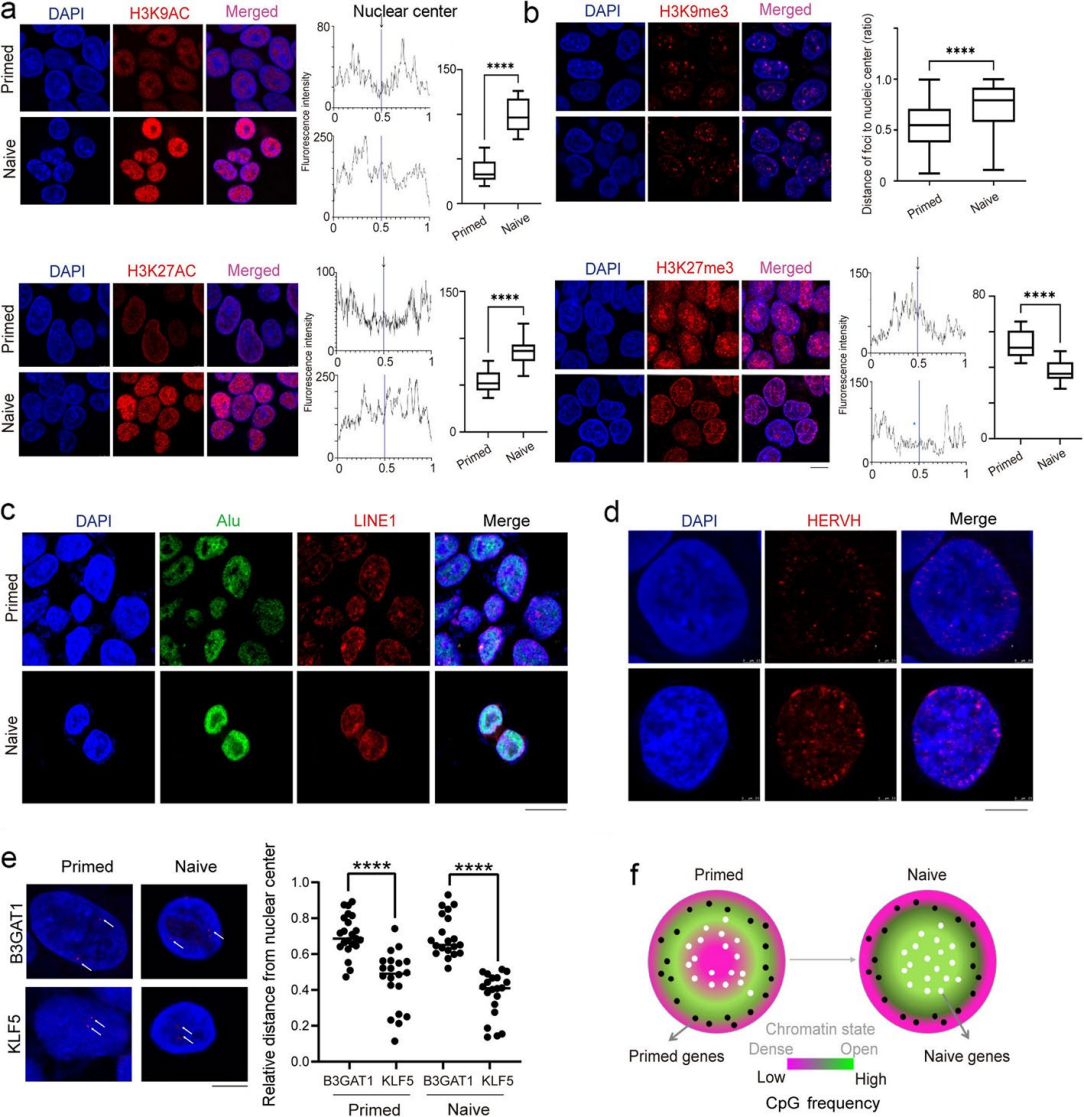
Distinct histone and chromatin distributions of naive and primed hESCs. **a, b** Histone distribution pattern of naive and primed hESCs, as detected by immunofluorescence. Nuclear distributions of H3K9ac, H3K27ac, H3K9me3, and H3K27me3 show differences in primed and naive hESCs (nuclei were stained by DAPI [blue]; scale bar, 10 μm). The ratios of the distances from H3K9me3 foci to the nuclear center were calculated by discerning approximately 130 signals from approximately 25 cellular nuclei, with a ratio = 0 indicating the center of a nucleus and a ratio = 1 indicating the edge of a nucleus. Average fluorescence intensity was calculated based on approximately 25 cell nuclei. Data shown are representative of three independent experiments with biological triplicates per experiment (data are presented as means ± standard deviation [SD]; *P* values were determined by a two-sided Student’s *t* test. *t*-test; ****P < 0.0001). **c** Representative images of Alu (green) and LINE1 (red) repeats, as revealed by DNA FISH in primed and naive cells (DNA was labeled by DAPI [blue]; scar bar, 20 μm). **d, e** Representative images of DNA FISH for the HERVH element, and B3GAT1 and KLF5 genomic regions (nuclei were stained with DAPI [blue]; scale bar, 5 μm). Experiments were repeated three times, with approximately 20 signals for 8–10 cells counted (*t* test. ****P < 0.0001; *P* values were determined by two-sided Student’s *t* test). **f** Proposed simplified model to illustrate differential genomic structure and compartmentalization and preferential localization of specific genes for naive and primed states in human ESCs. Naive genes in naive hESCs were principally localized to the active euchromatin compartment at the central nucleus, whereas heterochromatin was organized at the nuclear center of the primed state, suppressing naive genes. The active chromatin compartment at the nuclear center shows relaxed chromatin interactions that effectively facilitate enhancer activity with respect to the transcription of naive genes for naive pluripotency. This model provides insights into specific gene expression in association with chromatin structure and histone modifications

The aforementioned genomic structural organization, as determined by Dip-C and ATAC-seq, revealed that the central nuclear region of naive hESCs was characterized by active euchromatin and a relaxed chromatin structure for the transcription of naive pluripotent genes, whereas the nuclear center of primed hESCs was enriched with heterochromatic organization that suppressed naive pluripotent genes. The enhancers of genes can be marked by acetylation of histone H3 on lysine 27 (H3K27ac). Thus, to further confirm the genomic analysis data and to evaluate whether histone modifications also showed regional preference in the formed A–B compartments of naive hESCs relative to primed hESCs, we performed immunofluorescence microscopy to evaluate the distribution of the following relevant histone modifications: H3K9ac, H3K27ac, H3K9me3, and H3K27me3, which are indicative of constitutive and facultative heterochromatin, respectively. Both naive and primed hESCs expressed H3K9ac at high levels but exhibited distinctly different patterns (Fig. 7a, b and Additional file 1: Fig. S5a). Notably, while H3K9ac immunofluorescence in primed cells was missing at the larger center of a nucleus, with strong staining for heterochromatin by DAPI, the nuclear center was mostly enriched for H3K9ac in naive cells. H3K9ac-stained regions in primed cells coincided with chromatin localization and augmented expression of primed pluripotent genes (Additional file 1: Fig. S4a). Immunofluorescence of H3K27ac also showed differential distribution between naive and primed cells. H3K27ac was chiefly distributed at the nuclear edge of primed hESCs, forming a rosette distribution that was unlike its mostly even distribution for H3K9ac in the interior of the nucleus in naive hESCs (Fig. 7 and Additional file 1: Fig. S5b). H3K9me3 and H3K27me3 were also differentially expressed in the two states, and their foci were principally located at the nuclear center of primed cells. In naive cells, H3K9me3 was primarily distributed at the nuclear border, and H3K27me3 was dramatically reduced (also Additional file 1: Figs. S5c, S6a). H3K27ac was highly expressed in naive hESCs by western blot analysis, while H3K27me3 was highly expressed in primed hESCs (Additional file 1: Fig. S6b), supporting our immunofluorescence data.

Differences in the abundances and distributions of these histone modifications also coincided with differential chromatin structures, compartmentalization, and specific gene expression in naive and primed hESCs. The spatial separation of gene loci in the chromatin within the nucleus plays an important role in the regulation of cell fate, and it has been reported that the distribution of some transposable elements in the nucleus shows the characteristics of spatial separation (28). In mouse and human cells, the nuclear edge and the periphery of the nucleoli are enriched with *LINE1* sequences, and the interior of the nucleus is rich in *Alu* sequences. Compared with other subclasses of repeats and according to available Hi-C data, approximately 65% (80% in mouse cells) of SINE repeats (Alu in humans) are enriched in the A compartment and about 60% (80% in mouse cells) of L1 repeats (LINE1 in humans) are enriched in the B compartment. Homotypic clustering of L1 and B1 repeats characterizes and predicts compartmental organization [28] to a degree. We also determined the distribution of *LINE1* and *ALU* sequences in the nucleus in both pluripotent states (Fig. 7c and Additional file 1: Fig. S6c, d) and noted that LINE-1- orf-1p, a protein that plays an important role in the transposition of *LINE1*, was not expressed in naive cells but was normally expressed in primed cells (Additional file 1: Fig. S7a).

In addition, de novo insertion of *HERVH* elements in H9 primed cells introduces new TAD boundaries, and the transcriptional inhibition of *HERVH* elements prevents the formation of boundaries [29]. The transcripts of *HERVH* in primed cells are primarily distributed around the nucleus [13], and we detected the locations of *HERVH* elements in the nuclei of both cellular states. It is interesting to note that *HERVH* elements were not only distributed at the periphery of the nucleus but also in areas close to the center of the nucleus in naive cells relative to primed cells (Fig. 7d and Additional file 1: Fig. S7b).

To further validate whether the naive and primed states were regulated by different chromatin structures and compartmentalization in the nucleus, we used DNA FISH to determine the location of loci for the naive marker gene KLF5 and primed maker gene B3GAT1 in the nucleus. In both primed and naive cells, the position of the KLF5 locus was closer to the center of the nucleus than that of the B3GAT1 locus, and the position of the B3GAT1 locus was closer to the periphery of the nucleus (Fig. 7e and Additional file 1: Fig. S7c). In addition, the locations of the KLF5 locus and B3GAT1 locus in the nucleus did not change significantly in either state, implying that the gene expression regulation of KLF5 and B3GAT1 was to a degree dominated by the corresponding unique chromatin structures and compartmentalization.

Based on our data, we propose a novel model for pluripotent states whereby the primed state is maintained by the chromatin organization so as to accumulate heterochromatin at the nuclear center in order to suppress naive pluripotent genes and to form active euchromatin between the nuclear center and the border, facilitating the expression of the primed pluripotent genes. Following conversion to the extended naive pluripotent state, chromatin is then re-organized such that the active euchromatin is formed at the nuclear center, activating naive pluripotent genes; and the heterochromatic border nuclear region likely suppresses primed pluripotent genes (Fig. 7f). An organized reversal of the chromatin A–B compartment that enables chromatin accessibility in different nuclear regions and the presence of histone modifications thereby form the basis for differential gene expression in the two pluripotent states.

## Discussion

Our single-cell, structural 3D chromatin reconstruction revealed distinctive features of genomic organization and compartmentalization of pluripotent states. Specifically, spatial interactions of relaxed or flexible chromatin at the nuclear center provided structural bases for chromatin accessibility and the transcription of naive pluripotent genes in the naive state, facilitated by active histone modifications; whereas condensed heterochromatin arranged at the central nuclear region restricted chromatin accessibility and suppressed naive pluripotent genes in the primed state. Primed pluripotent genes were localized in the less-condensed chromatin region between the central heterochromatin and active chromatin at the nuclear periphery, and this organization enabled the expression of primed pluripotent genes (Fig. 7f). Previous studies found that a switch from active compartment A to repressive compartment B occurred primarily at the nuclear periphery during stem cell differentiation or reprogramming, or in primed and naive mouse stem cells [30]. By single-cell Dip-C, we ascertained that the inversion of inactive heterochromatin to active euchromatin or chromatin organization remodeling between primed and naive states of hESCs occurred at the nuclear center, which was quite compelling and had not been observed previously (Fig. 4a, b, e).

While acetylated histones (e.g., H3K27ac) are involved in regulating the transcription of pluripotent genes [31, 32], heterochromatin represented by H3K9me3 (which mediates chromatin compaction and compartmentalization [33, 34]) represses pluripotent genes and *HERVH* in the primed state [8]. Elevated levels of *HERVH* were also uncovered in naive vs. primed hESCs [14, 35]. How these histone marks are distributed at a genome-wide level, however, is not well understood. Our results showed that genomic organization exhibited a preferential localization or regional hotspot that recruited specific histones and that these histone modifications within specific chromatin structures regulated transcription so as to give rise to the naive or primed state. Transcription of *HERVH* in primed hESCs—despite being at lower levels than in naive hESCs—was detected by RNA-FISH in the chromatin region away from the nuclear center or at the nuclear periphery [13], and this region was consistent with the active chromatin structure in the primed state, as revealed in the present investigation. *HERVH* is also involved in creating TADs in hPSCs [29]. LTR7/HERVH sequences have long been hypothesized to exhibit enhancer activity, and they were originally proposed based on the enrichment of OCT4, NANOG, and SOX2 transcription factor binding sites specifically found in the LTR7 regions of HERVH elements [13]; *HERVH* relocation may result in the significantly increased protein expression of OCT4 and NANOG in naive hESCs (Additional file 1: Fig. S1c, S7b). Our data also revealed that the nuclear center became relaxed in naive cells—accompanied by *HERVH* relocation—suggesting that HERVH may be involved in regulating naive pluripotent genes. We speculate that *HERVH* plays critical roles in organizing euchromatin structure at the nuclear center in the naive state and at the nuclear periphery in the primed state. However, the technology that would allow the relocation of *HERVH* family genes in the cell nucleus is not yet available, and additional experiments are thus required to test this hypothesis in the future. Our data also support the concept that chromatin accessibility is associated with the enrichment of acetylated histones, while heterochromatin is linked to chromatin inaccessibility [36, 37]. Naive and primed hPSCs have also consistently been differentiated by ATAC-seq [22]. Furthermore, our data revealed that the chromatin structure was distinctly remodeled in a specific region at genome-wide levels in naive and primed states of human PSCs, allowing specific histone modifications to act spatially and effectively on the transcription of relevant genes.

Transcription is principally regulated by promoter–enhancer looping interactions mediated by CTCF between regulatory elements that are restricted within TADs [38], with TADs reflecting looping events or “loop domains” in the genome [39]. In addition, TADs may represent a “population average” of individual loops that differ on a cell-to-cell basis. It is acknowledged that TADs are indeed present in individual cells and that their observed hierarchy may reflect multimeric associations between individual regions within the TAD [39]. 3D chromosomal structures based on Hi-C chromosomal conformation capture data in population cells show that TADs are largely preserved during the transition between the naive and primed states of hESCs [40], and TAD structure is also revealed by single-cell Dip-C. While enhancers are occupied by transcription factors, mediators, and cohesin—and their associated nucleosomes marked by H3K27ac [40]—histone modifications themselves may not be required for chromatin organization in these differential states. Loss of H3K27ac, however, perturbs transcriptional but not 3D chromatin architectural resetting [31].

mESCs and mEpiSCs can be distinguished by active and inactive compartmental organization and switching in sub-nuclear positioning that is associated with replication timing [41], where it appears that the heterochromatin (B) compartment is located in the nuclear periphery, whereas the active euchromatin (A) compartment is in the interior in both naive mESCs and mEpiSCs. We herein showed that primed hESCs form inactive compartments enriched with heterochromatin at the nuclear center and active euchromatin at the nuclear periphery that are distinct from the active euchromatin and naive pluripotent genes localized at the nuclear center in naive hESCs. Naive pluripotent gene networks between human and mouse PSCs are not well conserved and more closely resemble their respective blastocysts [42]. In fact, the naive pluripotent state observed for mouse ESCs has been difficult to capture in hESCs, appearing to be transitory in the human embryo itself. Thus, the direct application of mouse embryology to humans has not always been successful due to fundamental developmental differences between the two species [43]. There were also some differences between the naive hESC lines derived directly from the early embryo [5, 44] and those that we employed in the present study. The naive cells we used in this work were derived from the conversion of the primed hESC line H9 by an LTR7-GFP reporter, and we maintained the HERVH hyper-activation required for human pluripotency. The expression profile of naive cells enriched using the HERVH reporter in our analysis most closely resembled the inner cell mass when compared with the naive cells obtained by Gafni et al. [5] and showed a similar XIST expression and H3K27me3 distribution pattern around the X chromosome [14]. However, we did not further assess the naive state by generating functional human–mouse chimeras or create and analyze an epigenetic signature that included DNA methylation and the deposition of H3K27me3.

In summary, our data provide insights into the chromatin structure in naive and primed states of hESCs. We identified specific patterns of changes in transcription and chromatin structure that might explain the genes differentially expressed between naive and primed hESCs. We integrated RNA-seq, ATAC-seq, and single-cell Dip-C data and selected a group of genes that were previously employed to mark naive and primed hESCs, yielding an association of gene transcription with chromatin openness and between chromatin structure and active compartmentalization. However, one limitation to our analysis was that the NGS sequencing data from ATAC-seq, RNA-seq, and Cut-tag-seq could not accurately distinguish between long reads coming from the paternal versus maternal parent. Only a single SNP or several SNPs in the fragments obtained by ATAC-seq RNA-seq or Cut&Tag-seq used in distinguishing parental alleles resulted in a high false-positive rate, and we could not derive allelic profiles of gene expression and chromatin configuration for the entire maternal and paternal chromosomal complements or even selected regions. We anticipate that the uncovered complexity of compartmentalized organization in regulating the pluripotent states of human PSCs will enable us to better control cell fate in future applications. The ability to rewire cells for pluripotency exerts a direct influence on the current hurdles and limitations related to the quality and characteristics of human PSCs [15]. We posit that a complete understanding of the defined genomic organization and higher order structure at high resolution will lead to enhanced strategies for controlling pluripotent states or cell-type transitions that will likely inform potential cell replacement therapy in regenerative medicine.

## Conclusions

By taking advantage of a single-cell, diploid chromatin conformation capture method termed Dip-C that reveals sufficiently high-resolution genomic structures, we demonstrated that the naive pluripotent state was regulated by active euchromatin compartmentalization and fewer chromatin interactions in the interior of the nucleus. A naive pluripotent state was achieved by remodeling the active euchromatin compartment with fewer chromatin interactions at the nuclear center. This unique genomic organization was linked to elevated chromatin accessibility on enhancers and thus contributed to the high expression levels of naive pluripotent genes localized to this region. In contrast, the primed state exhibited intermingled genomic organization. Active euchromatin and primed pluripotent genes were distributed at the nuclear periphery, and repressive heterochromatin was densely concentrated at the nuclear center, reducing chromatin accessibility and the transcription of naive genes. We expect that these findings will engender a fuller understanding of compartmentalized organization in regulating the pluripotent states of human PSCs.

## Methods

### Human embryonic stem cell culture

Naive hESCs (also termed pre-implantation epiblast-like hPSCs (prEpiSCs)) were derived from the LTR7-GFP hESC_H9 cell line. The basal culture medium (500 ml) was composed of 240 ml of DMEM/F12 (Thermo Fisher Scientific, catalog. no. 21331020), 240 ml of neurobasal medium (Thermo Fisher Scientific, catalog. no. 21103049), 2 mM L-glutamine (Thermo Fisher Scientific, catalog. no. 25030081), non-essential amino acids (Thermo Fisher Scientific, catalog. no. 11140050), 1% N2 supplement (Thermo Fisher Scientific, catalog. no. 17502048), 2% B27 supplement (Thermo Fisher Scientific, catalog. no. 12587010, minus vitamin A), 100 mg/ml vitamin C (Sigma, catalog no. A4403), 50 ng/ml bovine albumin fraction V (Thermo Scientific, cat. no. 15260–037), 20 ng/ml IL-6 (PeproTech, catalog. no. AF-200–06), 20 ng/ml sIL-6R (PeproTech, catalog. no. 200-06R), 0.1 mM β-mercaptoethanol (Thermo Fisher Scientific, catalog. no. 21985023), and Primocin (InvivoGen, catalog. no. ant-pm-2). The naive/prEpiSCs were cultured in advanced GIX medium containing basal culture medium plus chemical cocktail (0.2 μM trametinib, GSK112022; Selleck S2673), 0.3 μM PD0325901, 2.0 μM XAV939, and 0.5 μM Gö6983), with the medium changed every other day. The dome-shaped colonies were dissociated with Accutase (Thermo Fisher Scientific, catalog. no. A1110501) into single cells and then passaged onto new Matrigel- or feeder-coated plates every 3–4 days. The primed hESCs were cultured in Essential 8 medium (Gibco, USA, A1517001). The round-flat-shaped colonies were dissociated with EDTA (Invitrogen, 1,943,035) into small clumps and then passaged onto new Matrigel- or feeder-coated plates every 3–4 days. Cells were cultured in a hypoxic incubator under a humidified atmosphere of 5% O_2_ and 5% CO_2_ at 37 °C. All cell lines used in this study were authenticated and tested for *Mycoplasma* contamination.

### Chromatin conformation capture by Dip-C

Cells were fixed in 2% paraformaldehyde (PFA) in PBS or serum-free culture medium for 10 min with rotation. PFA was quenched to a final concentration of 0.127 M by adding 2 M glycine through a 0.2-μm filter and incubated on ice for 5 min. Cells were washed with ice-cold PBS, followed by centrifugation at 600 g for 5 min, and the pellets were stored at − 80 °C.

Cellular permeabilization and digestion were based on previously described methods [26]. Cells were permeabilized in 500 μL of ice-cold Hi-C lysis buffer (10 mM Tris, pH 8.0; 10 mM NaCl; 0.2% IGEPAL CA 630) and 100 μL of protease inhibitor (Sigma, P8340) for ≥ 15 min on ice, washed in cold Hi-C lysis buffer on ice (with centrifugation at 2500* g* for 5 min), and further permeabilized in 50 μL of 0.5% SDS at 62 °C for 10 min. The SDS was quenched by adding 145 μL of water and 25 μL of 10% Triton X-100 and incubated at 37 °C for 15 min with rotation. The cells were then digested by adding 25 μL of 10 × NEB Buffer 2 and 20 μL of 25 U/μL MboI (NEB, R0147M) and incubated overnight at 37 °C with rotation. On the second day, the cells were washed with 1 mL of ligation buffer (1 × T4 DNA ligase buffer, NEB B0202S) and 0.1 mg/mL BSA (NEB B9000S) and ligated in 1 mL of ligation buffer and 10 μL of 1 U/μL T4 DNA ligase (Life Tech, 15,224–025) at 16 °C for 20 h.

### Single-cell isolation by flow cytometry

The ligated cells (in ligation buffer) were filtered through a 40-μm cell strainer (Falcon) and sorted into 0.2-mL UV-irradiated, DNA low-bind tubes (MAXYMum Recovery, Axygen) containing lysis buffer using a FACSAria III flow cytometer (BD, 85-μm nozzle). The area-scaling factor was set, and forward scatter (FSC)-A and side scatter (SSC)-A were used to exclude large-sized cellular structures or debris, and scatter SSC-W was set to avoid contamination by doublets or triplets. Single cells were sorted into a PCR tube by applying the “1.0 drop single” sorting mode. The collected single cells were stored for several months at − 80 °C.

### Whole-genome amplification in Dip-C

Appreciable DNA contact information can be lost in traditional bulk Hi-C, but we greatly reduced any loss by inserting *n* different tags. As a result, only 1/*n* of input DNA contacts was lost in our study. We implemented META with *n* = 20 tags, and the sequences were treated according to previously described methods [20].

### Preparation of META reagents

Carrier ssDNA (used in the lysis buffer) was from a META 20 primer mix stored at − 20 °C, and the assembled transposome was used for the transposition mix. One strand of the transposon was 5'-/Phos/CTGTCTCTTATACACATCT-3', and the other strand was in the form of 5'-[META tag]-AGATGTGTATAAGAGACAG-3' [20]. Each of the oligonucleotides (IDT, China; purification using PAGE) was dissolved in 0.1 × TE to a final concentration of 100 μM. For each of the *n* = 20 META tags, the two strands were annealed at a final concentration of 5 μM each. Twenty annealed transposons were subsequently combined in equal volumes. EZ-Tn5 transposase was provided by Lucigen USA (TNP92110). The transposome was assembled at a final concentration of 1.25 μM dimer (2.5 μM monomer), diluted 1:10 (125 nM dimer or 250 nM monomer), aliquoted for single use, and stored at − 80 °C. The 20-primer mix (for PCR mix 1) was in the form of 5'-[META tag]-AGATGTGTATAAG-3'. Each oligo (IDT, purification by standard desalting) was dissolved in 0.1 × TE to a final concentration of 100 μM and mixed in equal volumes (100 μM in total, or 5 μM each) and stored at − 20 °C. The 40-primer mix (for PCR mix 2) was in the form of 5'- ACACTCTTTCCCTACACGACGCTCTTCCGATCT- [META tag] -AGATGTGTATAAG-3' for one side of the Illumina adapter and in the form of 5'-GACTGGAGTTCAGACGTGTGCTCTTCCGATCT -[META tag] -AGATGTGTATAAG- 3' for the other side. Each oligo (IDT, purification by PAGE) was dissolved in 0.1 × TE to a final concentration of 50 μM and combined with an equal volume (50 μM in total, or 1.25 μM each) and stored at − 20 °C.

### Cell lysis

Single cells were lysed in 3 μL META lysis buffer (20 mM Tris pH 8.0, 20 mM NaCl, 0.1% Triton X-100, 15 mM DTT, 1 mM EDTA, 1.5 mg/mL Qiagen protease, 0.5 μM carrier ssDNA) at 50 °C for 6 h, 65 °C for 12 h, 70 °C for 30 min. For recent lots of Qiagen protease, lysis may need to be shortened (for example 50 °C or 65 °C for 1 h, 70 °C for 15 min). Lysed cells could be stored at − 80 °C for a few months. Alternatively, single cells might be directly placed in empty tubes and stored at − 80 °C for longer times prior to addition of META lysis buffer.

### Whole-genome amplification

Lysate was transposed by addition of 5 μL transposition mix (leading to a final concentration of 10 mM TAPS at pH 8.5, 5 mM MgCl2, 8% PEG 8000, 1:2640 (0.5 nM dimer) META transposome) and incubation at 55 °C for 10 min. Transposases were removed by addition of 2 μL Stop Mix (1 μL 2 mg/mL Qiagen protease diluted in water, and 1 μL 0.5 M NaCl, 75 mM EDTA) and incubation at 50 °C for 40 min, and 70 °C for 20 min. Whole-genome amplification was performed by addition of 10 μL PCR Mix 1 (4 μL Q5 reaction buffer (NEB), 4 μL Q5 high GC enhancer (NEB), 0.5 μL 100 mM MgCl2, 0.5 μL 100 μM (total) META 20-primer Mix, 0.4 μL 10 mM (each) dNTP mix, 0.2 μL water, 0.2 μL 20 mg/mL BSA (NEB B9000S), 0.2 μL Q5 (NEB M0491S)) and incubation at 72 °C for 3 min, 98 °C for 20 s, 12 cycles of (98 °C for 10 s, 65 °C for 1 min, 72 °C for 2 min), and 65 °C for 5 min.

### Library preparation

Sequencing libraries were prepared by two additional PCR steps. In the first PCR step, previous primers were removed by addition of 0.5 μL 20 U/uL ExoI (NEB M0293S) and incubation at 37 °C for 30 min, 72 °C for 20 min. White precipitates might form at this step or at the following steps. PCR was performed by addition of 9.5 μL PCR Mix 2 (2 μL Q5 reaction buffer (NEB), 2 μL Q5 high GC enhancer (NEB), 3 μL 50 μM (total) META 40-primer Mix, 0.2 μL 10 mM (each) dNTP mix, 2.2 μL water, 0.1 μL Q5 (NEB M0491S)) and incubation at 98 °C for 30 s, 2 cycles of 98 °C for 10 s + 65 °C for 1 min + 72 °C for 2 min, and 65 °C for 5 min. In the second PCR step, primers were similarly removed by addition of 0.5 μL 20 U/μL ExoI (NEB M0293S) and incubation at 37 °C for 30 min, and 72 °C for 20 min. PCR was similarly performed by addition of 2.5 μL NEB Index Primer (NEB E7335S, E7500S, E7710S, E7730S) and 7 μL PCR Mix 3 (2 μL Q5 reaction buffer (NEB), 2 μL Q5 high GC enhancer (NEB), 2.5 μL NEB Universal Primer, 0.2 μL 10 mM (each) dNTP mix, 0.2 μL water, 0.1 μL Q5 (NEB M0491S)) and incubation at 98 °C for 30 s, 2 or more cycles of 98 °C for 10 s + 65 °C for 1 min + 72 °C for 2 min, and 65 °C for 5 min. Libraries could be pooled at this step or at any step afterwards. Libraries were purified by a DNA Clean and Concentrator-5 column (Zymo D4013) with 200 μL DNA Binding Buffer (a ratio of 1:5) and eluted in 25 μL 0.1 × TE. Size selection was performed with Ampure XP beads (Beckman Coulter, typically 0.65 ×).

### Sequencing

Libraries were sequenced with paired-end 250 bp reads on a HiSeq 2500 (Illumina). To avoid diversity issues (especially at the 19 bp right after the META tag), 20% PhiX was added. Raw sequencing outputs were 40–45 Gb per cell, corresponding to raw sequencing depths of 10–16 × .

### Statistical property of interchromosomal and long-range intrachromosomal contacts

In Dip-C, given that an interchromosomal contact joined coordinate *x* on one chromosome and *y* on another, the two contacting chromosomes might be seen as “tethered”at (*x*, *y*) and thus formed more contacts nearby. Such conditional properties were hidden in bulk Hi-C because interchromosomal contacts were highly stochastic. Dip-C gives a contact joining coordinate *x* (bp, called a “leg”) on one chromosome and *y* (bp, the other “leg”of the same contact) on another, that another contact joined *x* + Δ*x* and *y* + Δ*y*. Naively, if the two contacting chromosomes were “concatenated” at (*x*, *y*) and intermingled as if they were a single chromosome, the conditional probability density would be proportional to (Δ*x* + Δ*y*)^−1^—in other words, the inverse of the *L*1 norm of (Δ*x*, Δ*y*). However, the conditional probability density was approximately proportional to the inverse of the *L*^0.5^ distance (not a norm, also known as the Minkoski distance of order 0.5) of (Δ*x*, Δ*y*); in other words, *p*(Δ*x*, Δ*y*) ∝ (√Δ*x* + √Δ*y*)^−2^. Therefore, simultaneously large Δx and Δy were relatively disfavored, suggesting that two contacting chromosomes tended not to fully intermingle but rather protrude into each other. This empirical formula held across different genomic scales and for long-range intrachromosomal contacts.

Code is available on GitHub as a “dip-c” package (https://github.com/tanlongzhi/dip-c) referred on Tan et al. [20]. Starting from FASTQ files, 3D reconstruction consisted of the following steps, preprocessing → alignment → contact identification → artifact removal → haplotype imputation (2D) → [with replicates from here on] 3 rounds of 3D reconstruction at 100-kb resolution + haplotype imputation (3D) → 2 rounds of 3D reconstruction at 20-kb resolution + haplotype imputation (3D). Below is a brief description of each analysis step in the “dip-c”package: Preprocessing most reads followed a format of [META tag]-AGATGTGTATAAGAGACAG- [genomic DNA -CTGTCTCTTATACACATCT-[reverse complement of another META tag], although a small fraction harbored extra META adaptors (and very rarely, genomic DNA in between, which was discarded).

### Alignment

Reads were mapped by BWA-MEM [20] (version 0.7.15) with default parameters to the human reference genome GRCh37.

### Contact identification

From each read or read pair, all high-quality (mapping quality ≥ 20, edit distance per bp alignment ≤ 0.05) primary and supplementary alignments (in BWA-MEM, different parts of a single read can be locally aligned to different regions of the genome, producing one primary alignment and one or more supplementary alignments) were extracted as “segments”. If a segment overlapped with a phased SNP, a haplotype would be assigned if base quality ≥ 20. Chromatin contacts were identified as all pairs of segment end points—each end point.

### Haplotype imputation (2D)

In each round of imputation, contacts in an “evidence” set voted to impute unknown haplotypes of contacts in a “target” set. For each target contact, a list of compatible haplotype tuples was first enumerated. Each evidence contact would then vote for haplotype tuples from this list, if such contact fell within 10 Mb in L0.5 distance from the target contact and was compatible with one and only one haplotype tuple from the list. Imputation would occur if the winning haplotype tuple gathered ≥ 3 votes and ≥ 90% of all votes.

Special care was taken for intrachromosomal contacts because intrahomologous contacts were far more frequent than interhomologous contacts, especially at short ranges (small genomic separation). A target contact would be assumed intrahomologous without voting, if its two legs were separated by ≤ 10 Mb; otherwise, voting still occurred but a winning interhomologous vote would only be accepted if two legs were separated by ≥ 100 Mb. In addition, intrachromosomal contacts that had unknown haplotypes on both legs were not imputed.

One leg as both the target and the evidence sets was estimated for the contact location. Such imputation was repeated two more times, each time with previous results as the new evidence set. Results were subsequently cleaned by removal of isolated contacts (< 2 other contacts that had the same haplotypes within 10 Mb in *L*^0.5^ distance). Finally, cleaned results were used as the evidence set to impute a target set of all interchromosomal contacts that had unknown haplotypes on both legs.

### 3D reconstruction

Simulated annealing was performed by nuc_dynamics [20] (parameters: “-temps 20 -s 8 4 2 0.4 0.2 0.1” for 100-kb structures or “-temps 20 -s 8 4 2 0.4 0.2 0.1 0.04 0.02” for 20 kb structures) with minor modifications. First, the backbone energy function remained harmonic for large distances to reduce imputation errors. Second, removal of isolated contacts was skipped because it was already performed. Third, the output was in a simple “3D genome (3DG)” format (tab delimited: chromosome name, genomic coordinate (bp), *x*, *y*, *z*) because the original PDB format did not allow > 99,999 atoms. An example code was provided to convert 3DG to mmCIF for visualization in PyMol (run “set connect_mode, 4” before loading).

### Contact-based analysis

To rule out artifacts from the 3D modeling procedure, some of our conclusions were confirmed by contact-based, instead of 3D-structure-based, analysis. The single-cell chromatin compartment of each genomic bin was defined as the average CpG frequency (instead of A/B compartment in the previous study) of other bins (excluding self) that it contacted (“contacting CpG”) [20], weighted by the number of contacts. Haplotype-resolved contacts (raw contacts whose haplotypes were assigned through imputation) were used for diploid features, while both haplotype-resolved and raw contacts were used for cell-type features.

### Western blot

Cells were washed twice in PBS, collected, and lysed in cell lysis buffer on ice for 30 min and then sonicated for 1 min at 60 of amplitude at 2-s intervals. After centrifugation at 10,000* g* 4 °C for 10 min, supernatant was transferred into new tubes. The concentration of the protein sample was measured by bicinchoninic acid assay, and then protein samples were boiled in SDS sample buffer at 95 °C for 10 min; 3–10 μg total protein of each cell extract was resolved by 10% Acr-Bis SDS-PAGE and transferred to polyvinylidene difluoride membranes (PVDF, Millipore). Nonspecific binding was blocked by incubation in 5% skim milk in TBST at room temperature for 2 h. Blots were then probed with primary antibodies by overnight incubation at 4 °C with OCT4, NANOG, H3K27me3, H3K9me3, or H3K9ac. Immunoreactivity bands were then probed for 2 h at room temperature with the appropriate horseradish peroxidase (HRP)-conjugated secondary antibodies, goat anti-Rabbit IgG-HRP, or goat anti-Mouse IgG (H + L)/HRP. Protein bands were detected by Chemiluminescent HRP substrate (WBKLS0500, Millipore).

#### DNA FISH

DNA FISH was based on the method described [45], with slight modifications. Briefly, for the DNA probes of genes B3GAT1, KLF5, and HERVH, specific genomic regions were first amplified by PCR, and then the PCR products were digoxigenin-labeled (Sig ma-Aldrich, 11,093,088,910) by nick translation (Roche, 10,976,776,001) according to the manufacturer’s recommended protocol. DNA oligonucleotide probes for Line1 and Alu labeled with FAM or Cy5 were synthesized directly by the company, and the sequence information is attached in Additional file 2: Table S1. The length of the PCR products for the genomic loci of B3GAT1 and KLF5 was about 80–100 kb, and the length of HERVH was about 8 kb. All PCR primers are detailed in Additional file 3: Table S2.

DNA FISH of LINE1 and Alu was performed by using a previously described protocol [28], with minor modification. After approximately 100 ng of labeled probe, 6 µg of human Cot-1 (Invitrogen, 15,279,011) and 10 µg of salmon sperm DNA (Sigma, D7656-1ML) were precipitated, the DNA was resuspended in hybridization buffer (2XSSC, pH 7.4, 10% dextran, and 50% formamide). Cells were incubated on 10% Matri Gel-coated cover glass at 37℃ for approximately 24 h and were fixed with 4% formaldehyde at room temperature for 10 min, washed twice with 70% ethanol. Samples could be stored at 4℃ in 70% ethanol; then 0.1 M Tris–HCl for 10 min at room temperature, 0.1% PBS-Triton X-100 for 10 min at room temperature. After treated by 20% PBS-glycerol for 30 min at room temperature, samples were repeatedly freeze-thawed using liquid nitrogen for five times. And the samples were treated with 0.1 M HCl at room temperature for 30 min, RNAase (100ug/ml) digested at 37° for 30 min, permeabilized with 0.5% PBS-Triton X100 at room temperature for 30 min, and pre-hybridized with 50% formamide/2XSSC at room temperature for 30 min; the probes and samples were denatured at 80 °C for 10 min. After which the probe was quickly added to the sample, hybridized at 80 ℃ for 10 min on a heating block and then hybridized at 37℃ overnight in a humidity chamber. Afterward, we incubated the sample for 15–18 h at 37 °C. The next day, the coverslip was washed for 15 min at 45 °C in 50% formamide/2XSSC pre-warmed at 45 °C, while shaking, followed by two washes, 15 min at 60 °C in 0.2 × SSC pre-warmed at 60 °C and 15 min at 45° in 2XSSC. After a brief wash in 2XSSC at RT, sample was blocked with 4% BSA + 0.2% Tween in 4 × SSC at RT for 1 h, and incubated with anti-digoxigenin (Roche, 11,333,089,001) at 4° overnight. The next day, the Rabbit Anti-Sheep IgG (H + L)-TRITC was diluted at 1:200 and used to detect the digoxigenin-labeled probe, and samples were mounted with SLOWFADE GOLD ANTIFADE DAPI (Thermo Scientific, S36942). Images were captured on a Leica TCS SP8 confocal microscope, and using the software image J, the distance from the center of nucleus to the edge of nucleus and the probe was calculated to determine the position of the probe in the nucleus based on the ratio of the two distances.

### Xist RNA-FISH

Xist RNA-FISH was performed according to the manufacturer’s instructions (Biosearch Technologies). Growing cells were washed with PBS and fixed with 10% formaldehyde for 10 min at room temperature. After being washed twice with PBS, cells were immersed in 70% ethanol for at least 1 h at 4℃. Wash Buffer A (Biosearch Technologies, SMF-WA1-60) were added to cells and incubated at room temperature for 5 min. After incubation with the hybridization buffer (Biosearch Technologies, SMF-HB1-10) containing Xist RNA-FISH probe (Biosearch Technologies, SMF-2038–1) for 12–16 h at 37℃, cells were washed with Wash Buffer A and Wash Buffer B (Biosearch Technologies, SMF-WA1-20). Nuclei were stained with 0.5 mg/ml DAPI in Vectashield mounting medium. Fluorescence was detected and imaged using Leica TCS SP8 confocal microscope.

### Immunofluorescence microscopy

Cells were washed twice in PBS, fixed in freshly prepared 3.7% paraformaldehyde for 25 min on 4 °C, and permeabilized in 0.1% Triton X-100 in blocking solution (3% goat serum plus 0.1% BSA in PBS) for 25 min at room temperature, then washed once in PBS, and blocked in blocking solution for 2 h. Cells were incubated overnight at 4 °C with primary antibodies anti-H3K9me3 (Abcam, ab8898, 1:200 dilution), anti-H3K9ac (Abcam, ab32129, 1:200 dilution), anti-H3K27me3 (Millipore, 07–449, 1:200 dilution), H3K27ac (Abcam, ab177178, 1:200 dilution) in blocking solution. Cells were washed three times each for 15 min with blocking solution and incubated for 2 h with secondary antibodies at room temperature. Goat Anti-Rabbit IgG (H + L) Alexa Fluor® 594 (Jackson, 111–585-003, 1:200) was used in blocking solution. Samples were washed and counterstained with 0.5 mg/ml DAPI(Roche) in Vectashield mounting medium. Images were visualized and photographed using a Leica TCS SP8 confocal microscope. The distribution of fluorescence intensity of H3K9ac, H3K27ac, and H3K27me3 among the single nuclei and average fluorescence intensity are calculated by Leica LAS X.

### CUT&Tag

CUT&Tag was performed as previously described [46], with minor modifications. Briefly, 20,000 ~ 50,000 cells per sample replicate were washed in Wash Buffer [1 mL 1 M HEPES pH 7.5 (Sigma–Aldrich, H3375), 1.5 mL 5 M NaCl (Sigma–Aldrich, S5886-1 KG), 12.5 μL 2 M Spermidine (MCE, HY-B1776), Roche Complete Protease Inhibitor EDTA-Free tablet (Sigma–Aldrich, 4,693,132,001), and bring the final volume to 50 mL with dH2O], then immobilized on 10 µl of Concanavalin A-coated beads (Bangs Laboratories, BP531). Cells were cleared on a magnetic rack, then permeabilized with cold antibody buffer [20 mM HEPES pH 7.5, 150 mM NaCl, 2 mM EDTA, 0.1% BSA, 0.5 mM Spermidine and 1 × protease inhibitor cocktail containing 0.05% Digitonin (AbMole, M5020-100 mg)] on ice. The cells were then incubated with anti-H3K9me3 (Abcam, ab8898, 1:100 dilution), anti-H3K9ac (Abcam, ab32129, 1:100 dilution), anti-H3K27me3 (Millipore, 07–449, 1:100 dilution), H3K27ac (Abcam, ab177178, 1:100 dilution), and anti-CTCF (Millipore, ab128873,1:100 dilution) for 2 h at RT on a shaker. The primary antibody was cleared on a magnetic rack. Goat anti-Rabbit IgG secondary antibody (Millipore, 07–449) was diluted 1:100 in Dig-wash buffer (20 mM HEPES pH 7.5, 150 mM NaCl, 0.5 mM Spermidine, and 1 × protease inhibitor cocktail containing 0.05% Digitonin) and incubated at RT for an hour. Cells were cleared on a magnetic rack and washed three times with 700 µl of Dig-wash buffer. A 1:100 diluted of pA-Tn5 adapter complex combining adapter primers (Additional file 3: Table S2) and pA-Tn5 according to the manufacturer’s instructions (Vazyme, S603-01) was prepared in Dig-300 Buffer [20 mM HEPES pH 7.5, 300 mM NaCl, 0.5 mM Spermidine and 1 × protease inhibitor cocktail containing 0.05% Digitonin]. Cells were cleared on a magnetic rack and incubated by adding 100 ul of pA-Tn5 at RT for 1 h. Cells were washed with 700 µl of Dig-300 buffer, resuspended in 300 ul of Tagmentation buffer [10 mM MgCl2 in Dig-300 Buffer], and incubated at 37 °C for 1 h; 10 µl of 0.5 M EDTA, 3 µl of 10% SDS, and 2.5 µl of 20 mg/mL Proteinase K was added to each reaction to stop the tagmentation at 37 °C overnight. DNA was purified using phenol/chloroform/isoamyl alcohol (PCI) extraction followed by chloroform extraction and precipitated with glycogen and ethanol. DNA was pelleted with a high-speed spin at 4 °C, washed, air dried for 5 min, and resuspended in 25 µl of double-distilled water (ddH2O) containing 100 µg/ml RNase. The DNA was then PCR amplified using TruePrep Index Kit V4 for Illumina (Vazyme, TD204) and cleaned up with VAHTS DNA Clean Beads (Vazyme, N411-01). Then library quality was assessed on the Agilent Bioanalyzer 2100 system and the library preparations sequenced on an Illumina Hiseq platform.

### Library preparation and RNA-sequencing

mRNA was purified from total RNA using poly-T oligo-attached magnetic beads. Fragmentation was carried out using divalent cations under elevated temperature in NEB Next First Strand Synthesis Reaction Buffer (5 ×). First strand cDNA was synthesized using random hexamer primer and M-MLV Reverse Transcriptase (RNase H^−^). Second strand cDNA synthesis was subsequently performed using DNA Polymerase I and RNase H. Remaining overhangs were converted into blunt ends via exonuclease/polymerase activities. After adenylation of 3’ ends of DNA fragments, NEB Next Adaptor with hairpin loop structure were ligated to prepare for hybridization. To select cDNA fragments of preferentially 150 ~ 200 bp in length, the library fragments were purified with AMPure XP system (Beckman Coulter, Beverly, USA). Then 3 μl USER Enzyme (NEB, USA) was used with size-selected and cDNA adaptor ligated at 37 °C for 15 min followed by 5 min at 95 °C prior to PCR. PCR was performed with Phusion High-Fidelity DNA polymerase, Universal PCR primers, and Index Primer. At last, PCR products were purified using AMPure XP system and library quality assessed on the Agilent Bioanalyzer 2100 system. Cluster of the index-coded samples was performed on a cBot Cluster Generation System using TruSeq PE Cluster Kit (Illumina) according to the manufacturer’s instructions. After cluster generation, the library preparations were sequenced on an Illumina Hiseq platform.

### Bioinformatics analysis for differentially expressed genes

The adapter sequences of RNA-seq data and low-quality reads with Phred score < 5 were deleted with Cutadapt before further processing. H19 was used to map RNA-seq data to mm10 with parameter—k 20. Genes were annotated according to the Ensembl database. Transposable element annotations were obtained from UCSC Genome Browser (RepeatMasker). Reads were counted using featureCounts. The resulting matrix of read counts was then loaded into RStudio (R version 3.4.2), and DESeq2 used to identify differentially expressed genes with expression fold change > 2 and adjusted *p* value < 0.01. Functional enrichment (GO annotation or KEGG) of gene sets with different expression patterns was performed using clusterProfiler. Genes with expression fold change > 2 and adjusted *p* value < 0.01 according to DEseq2 were used for GO, KEGG, and Gene set enrichment analysis (GSEA) analysis by clusterProfiler**.**

### ATAC-seq

The assay for transposase-accessible chromatin using sequencing (ATAC-seq) was basically based on the procedure described [47]. Cells were harvested and counted. Number of 500 ~ 1000 cells were used for ATAC-seq. The number of cells at this step is crucial, as the transposase-to-cell ratio determines the distribution of DNA fragments generated. Sample with 500 ~ 1000 cells was centrifuged for 5 min at 500 × *g* at 4 °C. Supernatant was removed and discarded. Cells were washed once with 50 μl of cold PBS buffer, centrifuged for 5 min at 500 × at 4 °C, and supernatant removed and discarded. The cell pellet in 50 μl of cold lysis buffer was resuspended by gently pipetting up and down and lysed for 10 min on ice, followed by centrifugation immediately for 10 min at 500 × *g* at 4 °C. The supernatant was discarded and immediately continued to transposition reaction. Lysis buffer used was 10 mM Tris–HCl (pH 7.4), 10 mM NaCl, 3 mM MgCl_2_, and 0.5% NP-40.

For transposition reaction and purification, TruePrep® DNA Libraryaf Prep Kit V2 for Illumina (Vazyme, TD502, 5 ng) for tagmentation was used. After tagmentation reaction, AMP XP beads for purifying was used. In preparation, AMPure XP beads were equilibrated at room temperature for 30 min and mixed well; 25 μl (0.6 ×) beads were pipetted into the 50 μl PCR product, gently pipetted and mixed, and incubated at room temperature for 5 min (to remove long fragments). The reaction tube was centrifuged briefly and placed on a magnetic stand. After clarification, the supernatant (75 μl) was transferred to a new tube (be careful to mark the tube cap and tube body), and the magnetic beads discarded; 60 μl (1.2 ×) beads were pipetted into the supernatant, mixed by pipetting, and incubated at room temperature for 5 min (magnetic beads can be added to the new EP tube during the first incubation). The reaction tube was centrifuged briefly and placed on a magnetic stand. After clarification, the supernatant was removed, the tube was kept on the magnetic stand; 200 μl of freshly prepared 80% ethanol was added to rinse the magnetic beads, incubated at room temperature for 30 s, and the supernatant carefully removed. The rinse was repeated three times. The tube was kept on the magnetic stand, the lid left open and dry (not too dry, no alcohol residue at the bottom of the tube). The tube was taken out of the magnetic stand, 25 μl of sterile ultrapure water added, the magnetic beads smashed from the wall, mixed well, and incubated at room temperature for 5 min. The reaction tube was centrifuged briefly and placed on a magnetic stand. After clarification, 20 μl of supernatant was carefully transferred to a new tube and stored at − 20 °C.

Library was constructed as follow. TruePrep TM Index V4 for Illumina (Vazyme TD204) was used to enrich DNA and PCR amplification. The PCR products were purified with VAHTS DNA Clean Beads (Vazyme N411). At last, DNA fragments of preferentially about 300 bp in length were selected, the library fragments were purified with AMPure XP system (Beckman Coulter, Beverly, USA), then library quality was assessed on the Agilent Bioanalyzer 2100 system and the library preparations sequenced on an Illumina Hiseq platform.

### ATAC-seq and CUT&Tag-seq data analysis

ATAC-seq: ChiLin pipeline 2.0.0 [48] (Qin et al. 2016) is used for QC and preprocess of the ATAC-seq. We use Burrows–Wheeler Aligner (BWA) [49] as a read mapping tool, and Model-based Analysis of ChIP-Seq (MACS2) [50] as a peak caller, with a *q*-value (FDR) threshold of 0.01. Based on a dynamic Poisson distribution, MACS2 can effectively capture local biases in the genome sequence, allowing for more sensitive and robust prediction of binding sites. Unique read for a position for peak calling is used to reduce false positive peaks, statically significant peaks are finally selected by calculated false discovery rate of reported peaks. Deeptools [51] is used for the heatmap plots. ATAC-seq peaks from all study samples were merged to create a union set of sites. Read densities were calculated for each peak for each sample, differential peaks between Naive and Primed were identified by DEseq2 [52] with adjusted *P* ≤ 0.05, |log2fold change|≥ 1.

Cut-tag: use FastQC v0.11.9 for quality control of raw sequencing readings. Using TrimGalore v0.6.6 to remove raw readings from low-quality base and linker sequences(https://github.com/FelixKrueger/TrimGalore). Compare the filtered reading with the reference mouse genome assembly mm10 of the mouse sample and the human genome assembly GRCh38 of the human sample using Bowtie2 v2.4.4. The options are end-to-end, very sensitive, no mixing, no inconsistency, phred33-I 10-X 700. Use the sorting function of samtools v1.13 to sort aligned bam files based on chromosome coordinates. Use the genomecov function of bedtools v2.30 to summarize the sorted bam files into a bedgraph file (Quinlan et al., 2010) [53]. In the case of samples with multiple biological replicates, use the unionBeg function combination of bedtools v2.30 to replicate specific bed chart files. In strict mode, perform peak calls on all bedgraph files using SEACR v1.3 by selecting the top 1% of the call peak. SEACR is specifically developed for CUT&RUN and is also a recommended pipeline for chromatin analysis data with very low background, such as CUT&Tag. Perform visual QC on peak values of bam files and calls using Seqmonk [54].

### Supplementary Information

Additional file 1: Fig S1. Transcriptome of naive and primed hESCs. Fig S2. Simulation of 3D structure of single-cell chromatin in naive and primed hESCs. Fig S3. Distinct 3D structures of chromosomes in naive and primed hESCs. Fig S4. Features of localization of marker gene locus and regulation in primed and naive hESCs. Fig S5. Fluorescence intensity distribution of epigenetic histone modifications in the nucleus. Fig S6. Distribution of epigenetic histone modifications and LINE1/Alu in primed and naive hESCs. Fig S7. Genomic localization of marker genes, HERVH, and expression of LINE-1-orf-1p in primed and naive hESCs. Fig S8. Radial positioning along the genome of primed or naive genes and enhancer in naive or primed hESCs. Fig S9. Uncropped scans of Western blot.

Additional file 2: Table S1. The sequencing information.

Additional file 3: Table S2. Gene List and Primer Information related the manuscript.

Additional file 4. Review history.

## Data Availability

Raw sequencing data reported in this paper were uploaded into NCBI with accession number PRJNA739271 [55]. And all the RNA-seq, CUT&Tag-seq and ATAC-seq processed data are available from Gene Expression Omnibus under accession number GEO: GSE260995 [56]. The bulk HiC data used in Fig. 4f are available from Genome Sequence Archive with the accession number CRA000852 [57]. The method to distinguish A/B parts in Fig. 4e is refereed to Lieberman Aiden [23]. No other scripts and software were used other than those mentioned in the “ Methods” section. All cell lines used in this study have been authenticated and are available upon request.
