# Supplementary material for: Single-cell 3D genome structure reveals distinct human pluripotent states
Source: Genome Biol. 2024 May 13;25:122. doi: 10.1186/s13059-024-03268-w (PMC11089717; doi:10.1186/s13059-024-03268-w)
Supplement: Supplementary file 3 — Additional file 3: Table S2. Gene List and Primer Information related the manuscript. [file 13059_2024_3268_MOESM3_ESM.docx]

**Supplemental Information**

**Additional file 1**: **Fig S1.** Transcriptome of naive and primed hESCs. **Fig S2.** Simulation of 3D structure of single-cell chromatin in naive and primed hESCs. **Fig S3.** Distinct 3D structures of chromosomes in naive and primed hESCs. **Fig S4.** Features of localization of marker gene locus and regulation in primed and naive hESCs. **Fig S5.** Fluorescence intensity distribution of epigenetic histone modifications in the nucleus. **Fig S6.** Distribution of epigenetic histone modifications and LINE1/Alu in primed and naive hESCs. **Fig S7.** Genomic localization of marker genes, HERVH, and expression of LINE-1-orf-1p in primed and naive hESCs. **Fig S8.** Radial positioning along the genome of primed or naive genes and enhancer in naive or primed hESCs. **Fig S9**. Uncropped scans of Western blot with molecular weight markers.

**Additional file 2:** **Table S1. The sequencing information.**

**Additional file 3: Table S2. Gene List and Primer Information related the manuscript.**


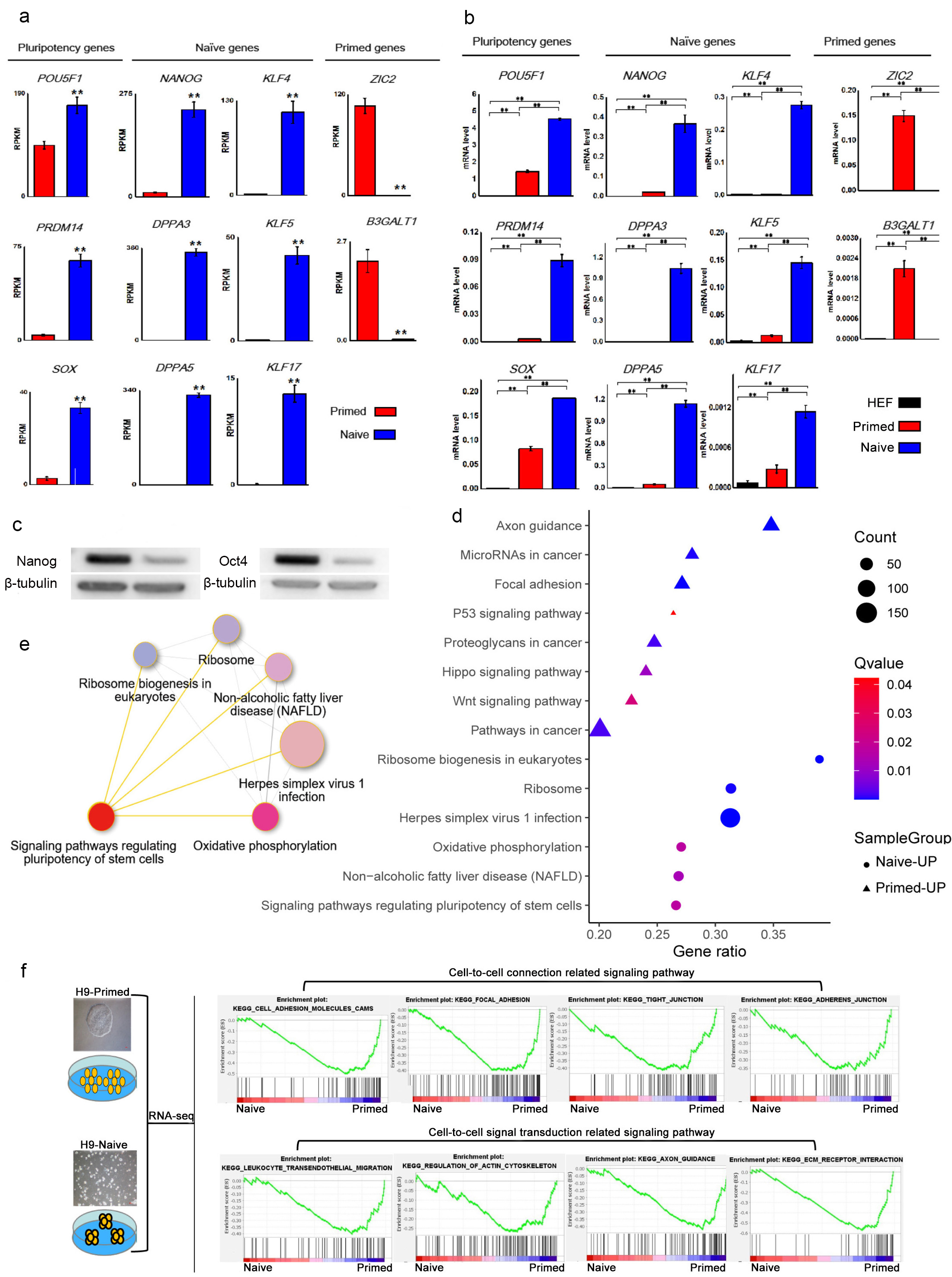


**Fig S1. Transcriptome of naive and primed hESCs.**

**a**, Expression levels (FPKMs) of pluripotency and corresponding marker genes in naïve and primed hESCs by RNA-seq. Data are presented as means ± standard deviation (SD). t-test **P <0.01. **b**, mRNA expression of pluripotency and corresponding marker genes in naïve and primed hESCs by Quantitative real-time q-PCR. Data are presented as means ± standard deviation (SD). t-test **P <0.01. **c**, Western blot analysis of protein levels of pluripotency genes. Experiments were repeated at least twice, with 2-3 biological repeats. **P<0.01. **d**, KEGG analysis of the pathways up-regulated in naive and primed hESCs from RNA-seq data. **e**, Relationship between enriched pathways up-regulated in naive state relative to primed state. **f**, GSEA enrichment plots showing that compared to primed cells, up-regulated genes in naïve cells are enriched in cell-to-cell connection and signal transduction related to signaling pathway.


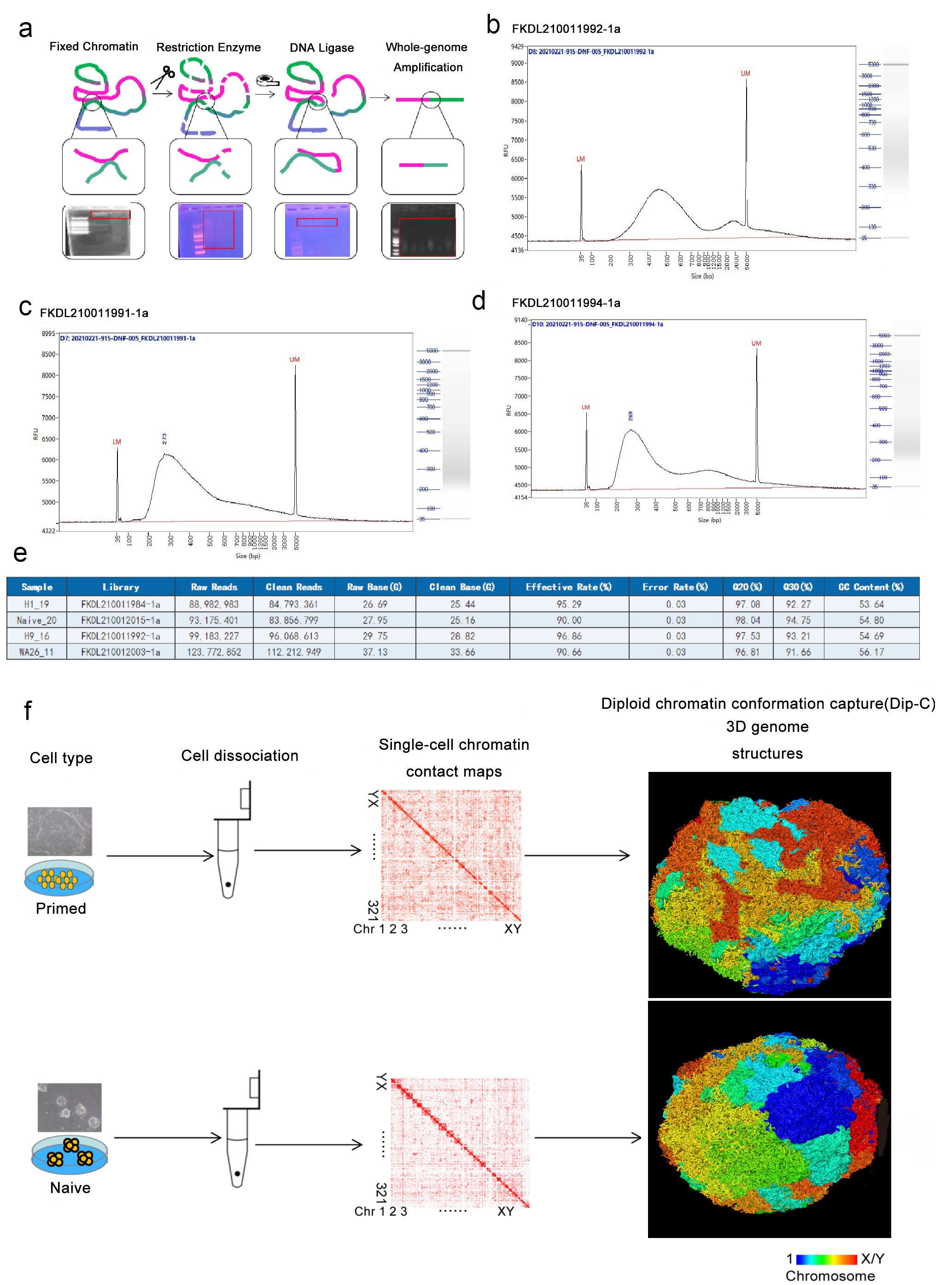


**Fig S2. Simulation of 3D structure of single-cell chromatin in naive and primed hESCs.**

**a**, Schematic diagram of the Dip-C chromatin conformation capture protocol and the test results of each step. 3D information of chromatin structure was encoded in the linear genome through proximity ligation of chromatin fragments, and the ligation product was then amplified by META (20) and sequenced**. b-d**, Fragment length distribution after whole-genome amplification by performing Bioanalyzer trace of a representative pooled library. Only desired products >300bp remained. **e,** Summary of Dip-C library quality. **f**, Process of Dip-C to simulate the chromatin structure in a single cell. Naive or primed human ESCs were sorted into single cells. After constructing Dip-C library, single cell chromatin contact map is created and 3D genome structure is simulated based on the library. Each experiment contained two biological repeats. The 3D chromatin structure is based on single-cell Diploid Chromatin Conformation Capture (Dip-C) data obtained from naive and primed hESCs. In primed hESCs, chromosomes are intermingled and show more chromatin interactions, whereas chromosomes are regionally localized and have less chromatin interactions in naive hESCs. Different colors represent different chromosomes (1,2,3,...X/Y).

**
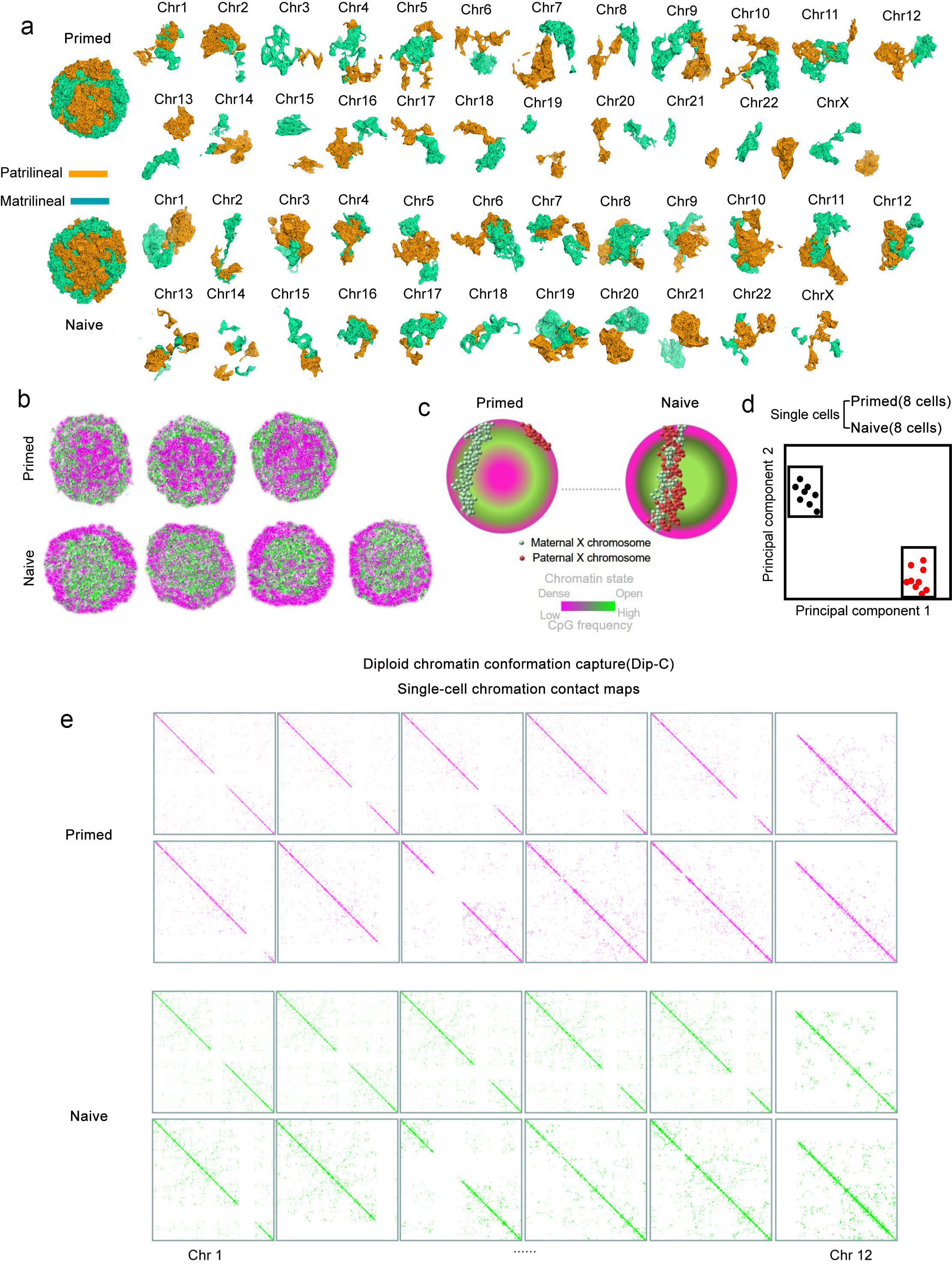
**

**Fig S3. Distinct 3D structures of chromosomes in naive and primed hESCs.**

**a**, Structural difference of all homologous Chromosomes from the parents between naïve and primed hESCs. **b**, Cross sections of other primed hESCs and naive hESCs, colored by CpG frequency. Each particle represents 40 kb of chromatin (~60 nm in radius). **c**, Schematic model of localization of X chromosome from the parents in naive and primed hESCs. Shrinkage paternal X chromosome is embedded within the cell surface, and the activated maternal chromosome X interludes from the nucleus in primed cells**. d**, Unsupervised clustering via PCA of single-cell chromatin compartment values showing two significantly different clusters (black, Naive hESCs; red, Primed hESCs). For each genomic locus (1 Mb bin size), the chromatin compartment value is defined as the average CpG frequency of all the loci that it contacts, and then rank-normalized to 0–1 in each cell. **e,** Average haplotype-resolved single-cell chromatin contact maps of 2D structure of chromosomes 1-12 in primed and naive hESCs. Each contact map was binned every 1 Mb and normalized by the mean (whose value was defined as one) of all bins.


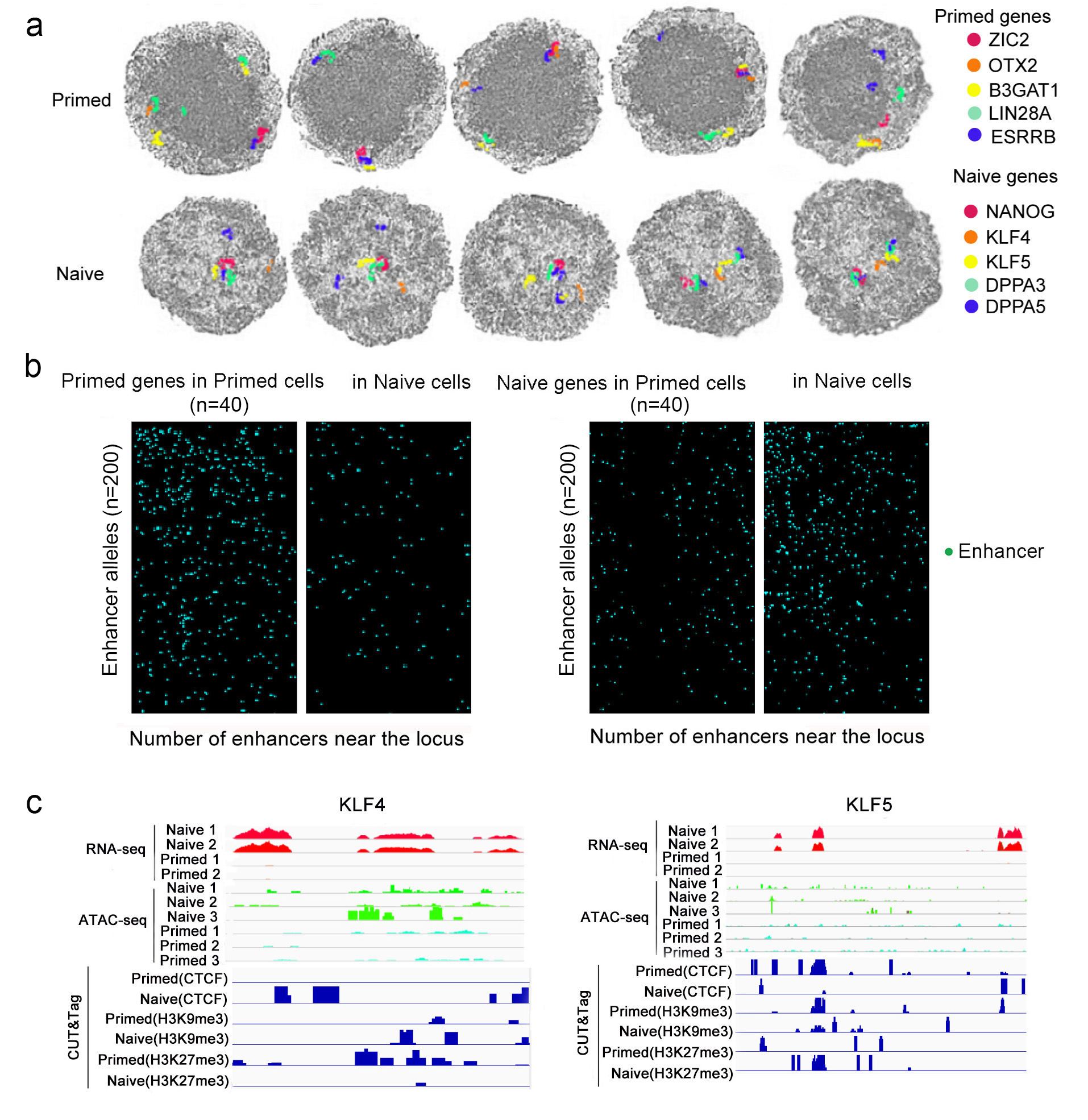


**Fig S4. Features of localization of marker gene locus and regulation in primed and naive hESCs.**

**a,** Chromatic localization of primed or naive genes. In naive state, naive genes (representative five marker genes are shown in different color) mainly are localized in the nuclear center whereby active euchromatin is organized. In primed cells, primed genes (representative five marker genes are shown in different color) are notably distributed between the nuclear periphery and the center, where euchromatin is accumulated. **b**, Number of enhancers of naive genes and primed genes in naive and primed hESCs. In each heat map, rows and columns are sorted by their average values. We selected the top 20 genes with high expression levels in Primed cells, including their alleles. The horizontal axis shows 40 (20x2) gene loci. The vertical axis represents the possible occurrence of enhancers at these gene loci. The top 100 enhancers with the highest probability of occurrence were selected, and the calculated allele loci were 200 (100x2). Blue dots appeared, indicating the possible occurrence of enhancers at that gene position (left). We selected the top 20 genes with high expression levels in Naive cells, including their alleles. The horizontal axis shows 40 (20x2) gene loci. The vertical axis represents the possible occurrence of enhancers at these gene loci. The top 100 enhancers with the highest probability of occurrence were selected, and the calculated allele loci were 200 (100x2) (Right). Blue dots appeared, indicating the possible occurrence of enhancers at that gene position. **c**, Joint analysis by ATAC-seq, CUT&Tag and RNA-seq of the relationships among gene expression, chromatin openness and epigenetic modifications in primed and naive cells. *KLF4* and *KLF5* are naive genes that are highly expressed in naive state hESCs compared to primed state, and show more chromatin openness. Data from two to three biological repeated experiments.


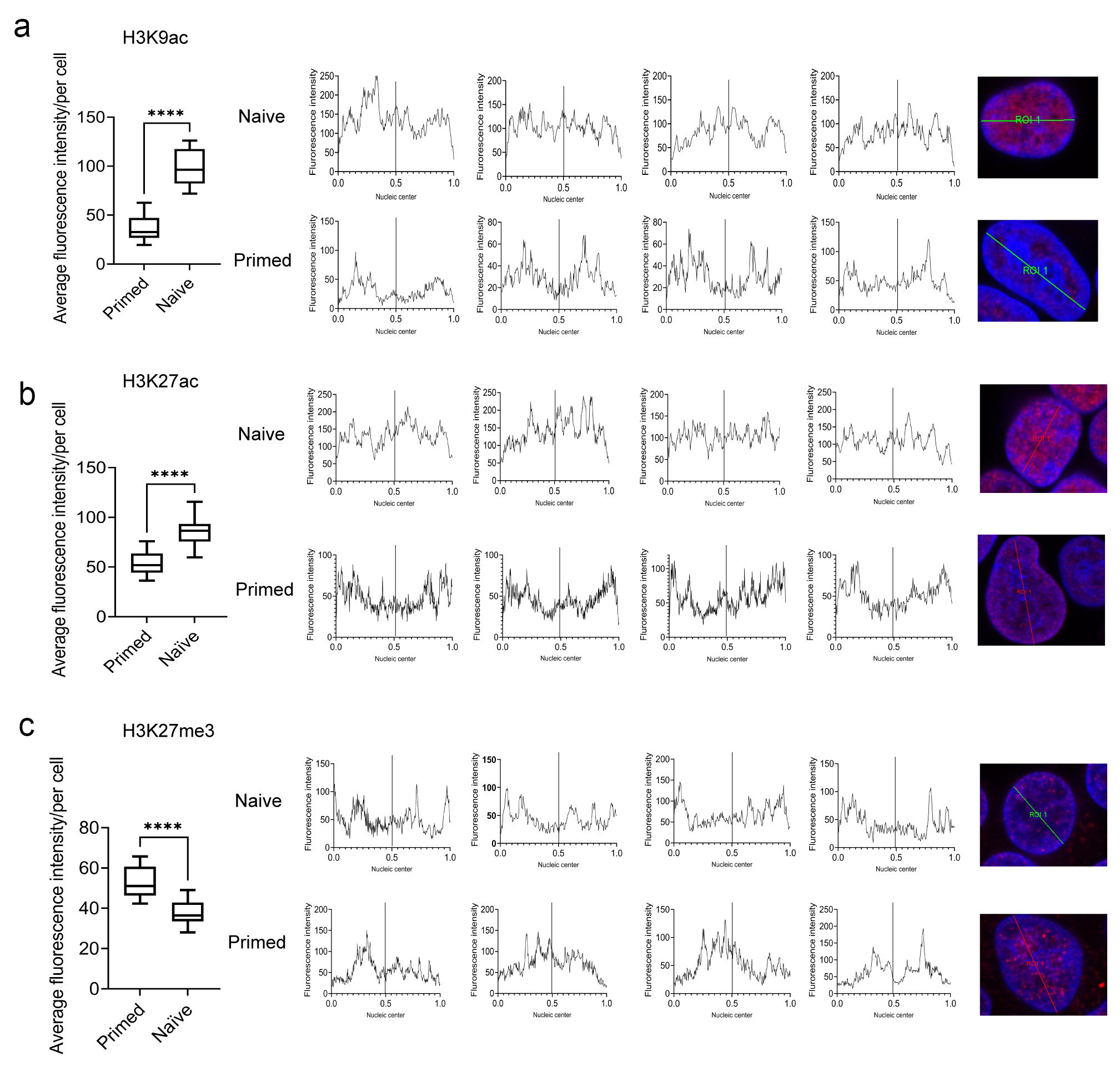


**Fig S5. Fluorescence intensity distribution of epigenetic histone modifications in the nucleus.**

**a, b, c,** Graph on the left is a count of the average fluorescence intensity for approximately 25 nuclei. Data are presented as means ± standard deviation (SD) and the peak-shaped graph in the middle is the distribution of fluorescence intensity for each region in the nucleus. Representative schematic of distribution of fluorescence intensity in the single nucleus is shown on the right. A line is drawn through the center of the nucleus. The vertical coordinate is the fluorescence intensity of each point on the line, and the horizontal coordinate is the ratio of the distance of each point on the line to the length of the line. ratio=0 and ratio=1 represent the two points at the edge of the nucleus, and ratio=0.5 represents the center of the nucleus. Data shown are representative of three independent experiments with biological triplicates per experiment. Data are presented as means ± standard deviation (SD). t-test ****P <0.0001.


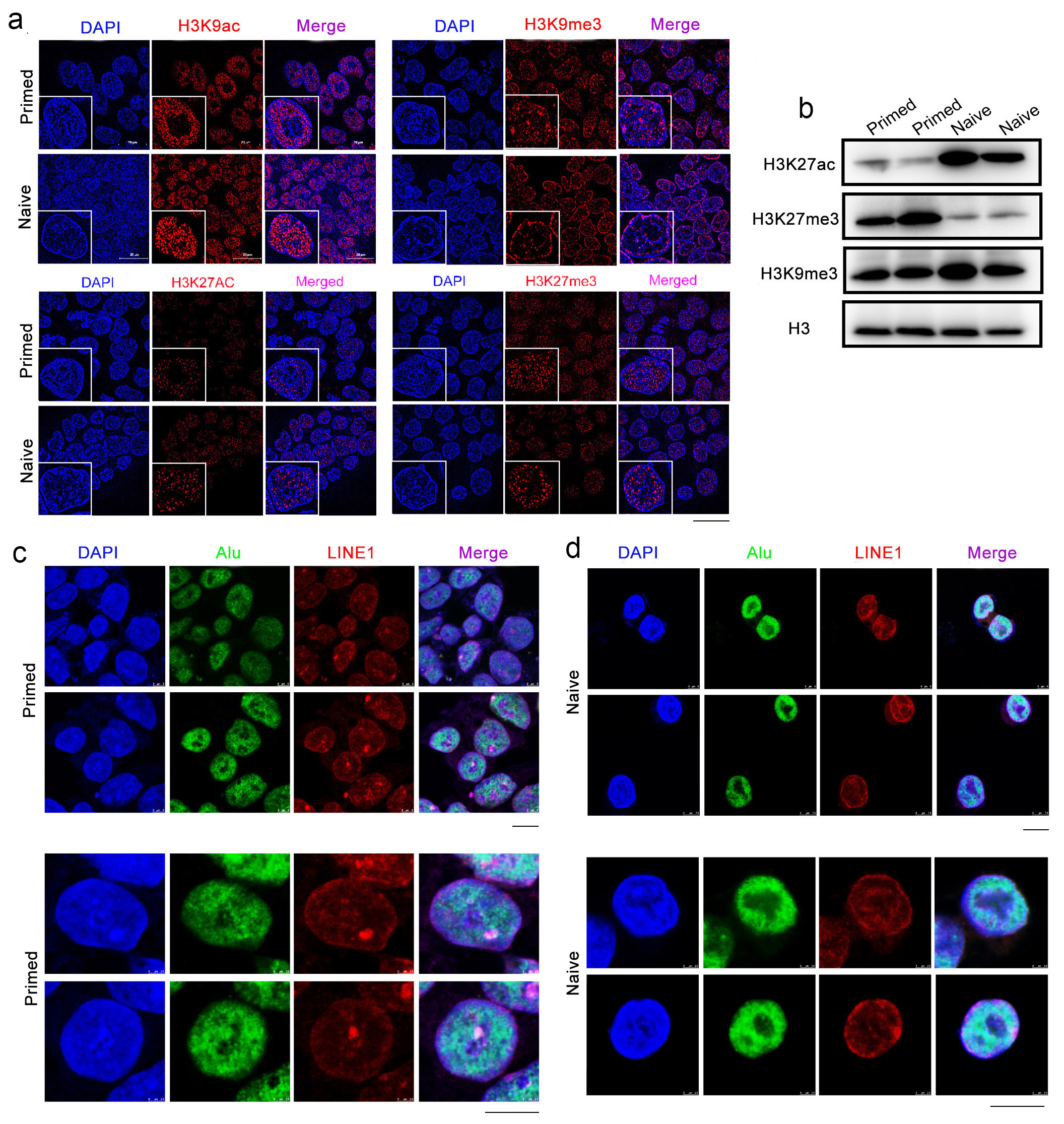


**Fig S6. Distribution of epigenetic histone modifications and LINE1/Alu in primed and naive hESCs.**

**a,** Immunofluorescence of histone distribution in naive and primed hESCs. Images were visualized and photographed using a Leica structure illumination microscope. Scale bar, 10 μm. **b**, Western blot analysis the protein levels of the histones. **c**, **d**, Representative images of Alu (green) and LINE1 (red) repeats revealed by DNA FISH in primed and naive hESCs. DNA is labeled by DAPI (blue). Images were visualized and photographed using Leica TCS. Scale bar, 10 μm.

**
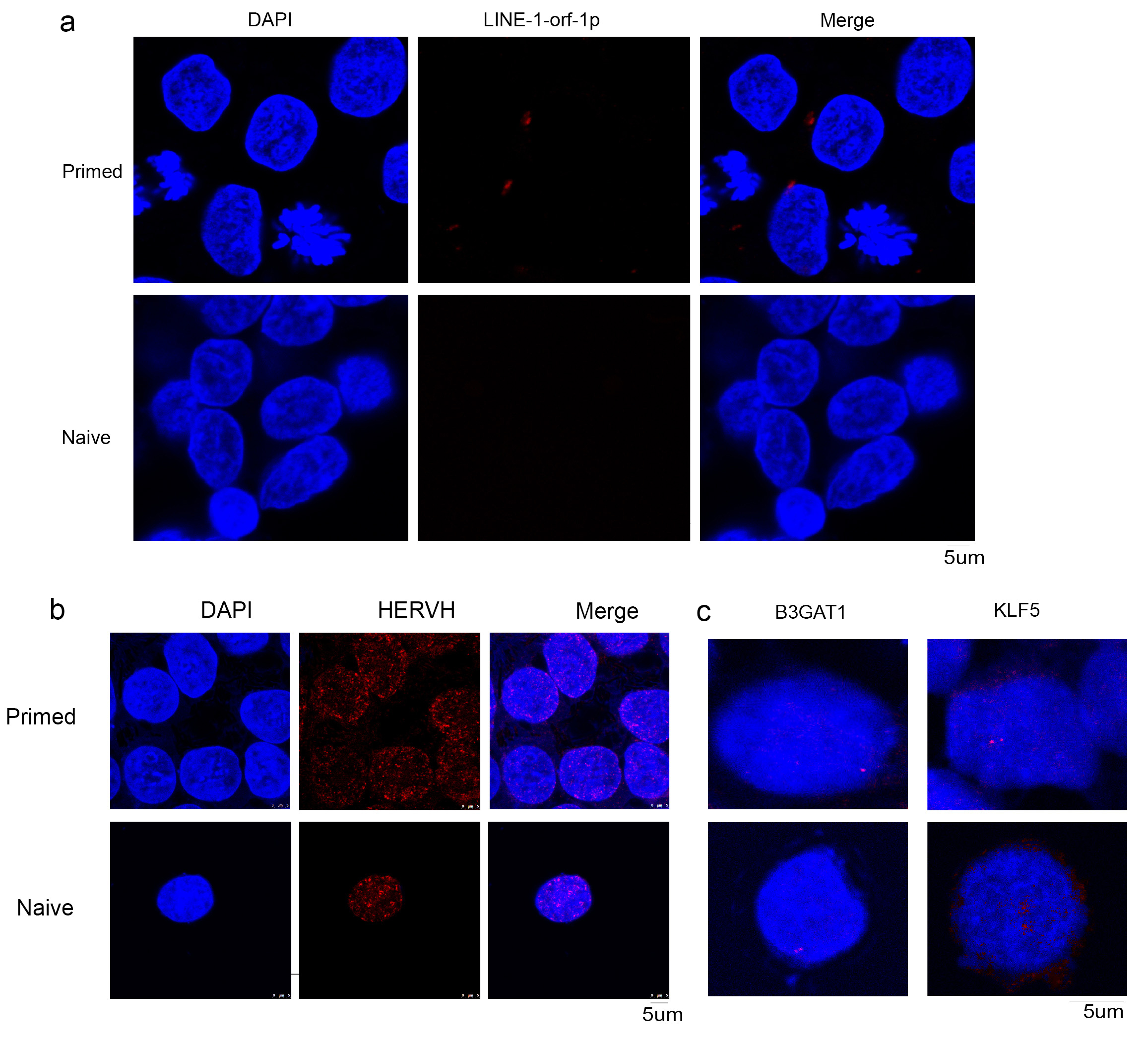
**

**Fig S7. Genomic localization of marker genes, HERVH, and expression of LINE-1-orf-1p in primed and naive hESCs.**

**a,** Immunofluorescence of the LINE-1-orf-1p (Red) in naive and primed hESCs. Scale bar, 10 μm. **b,** DNA FISH of HERVH in primed and naive cells. Nuclei were stained with DAPI (blue). Scale bar, 5 μm. **c**, DNA FISH using probes for the B3GAT1 and KLF5 in the genomic region in primed and naive cells. Scale bar, 5 μm. Images were visualized and photographed using Leica TCS SP8.


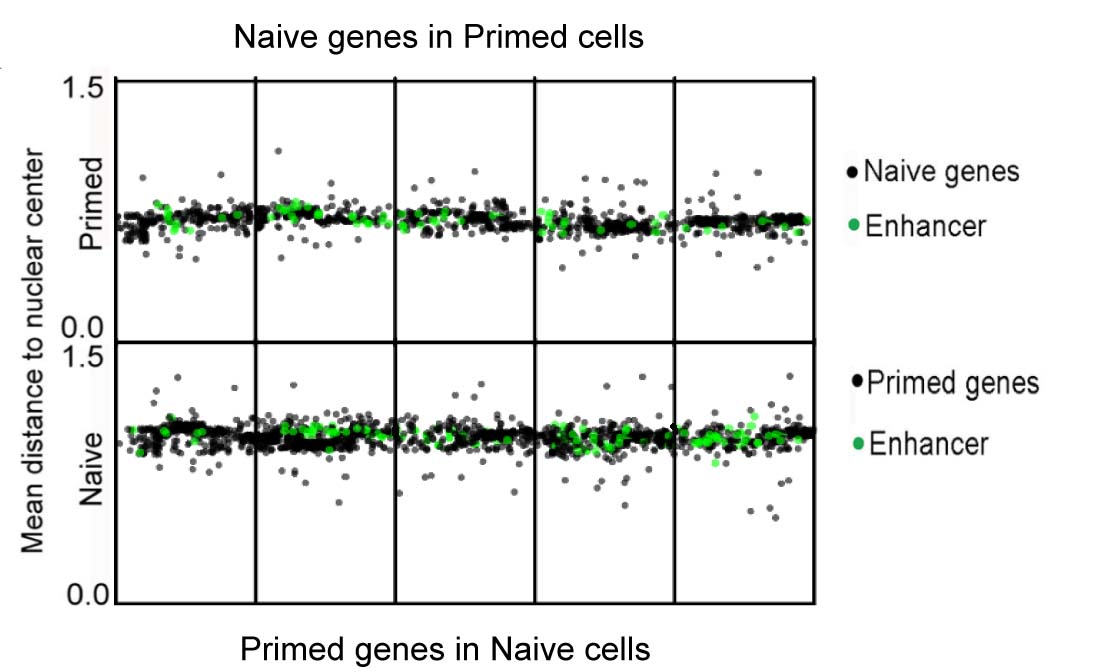


**Fig S8. Radial positioning along the genome of primed or naive genes and enhancer in naive or primed hESCs.**

**
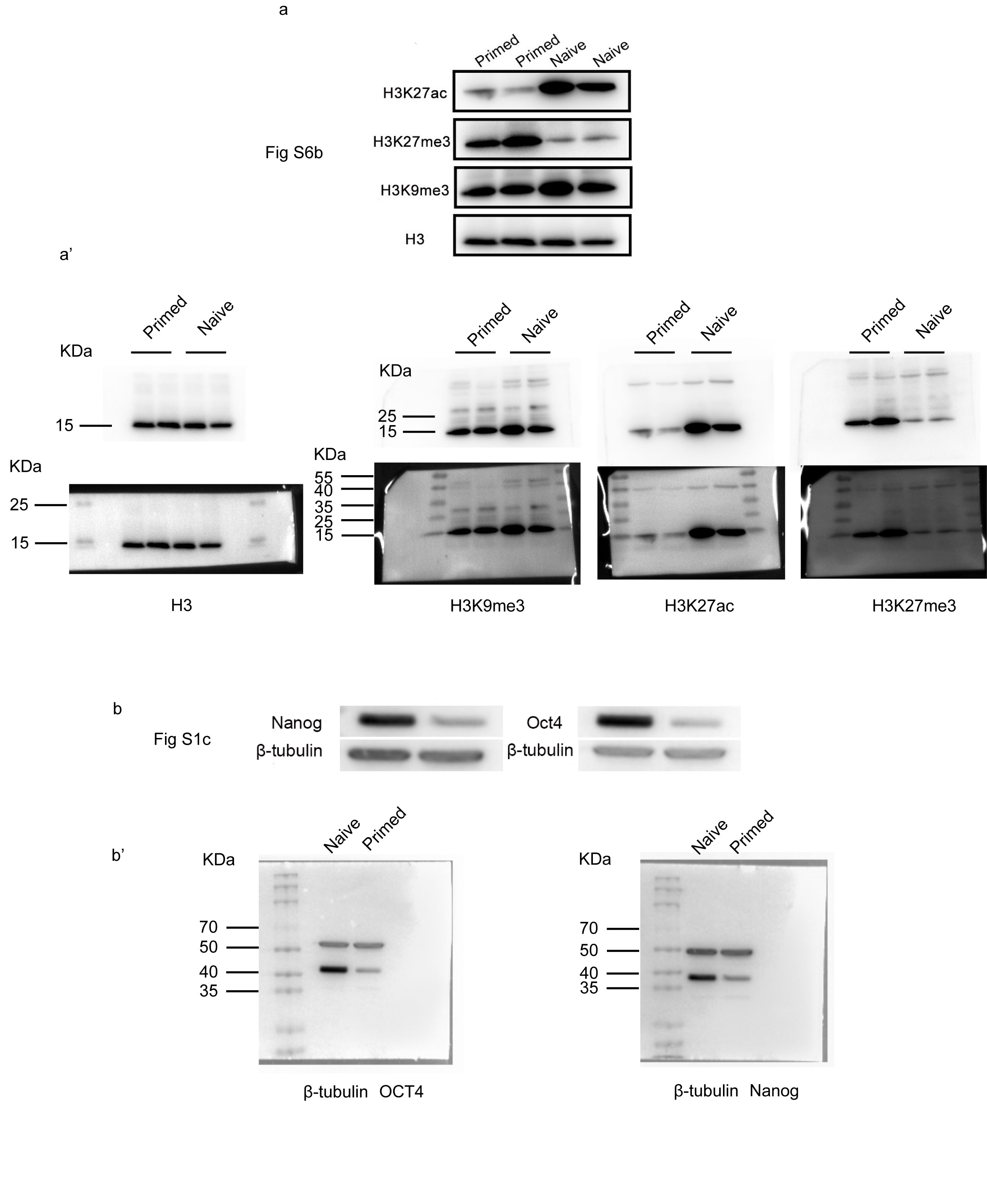
**

**Fig S9 Uncropped scans of Western blot.**

(a and a’). The uncropped Western blot for Fig S6d comparing the H3K9me3, H3K72me3 and H3K27ac protein level in naïve and primed hPSCs. H3 served as loading control. (a’) was the same gel as the image of (a) but with protein markers under bright field, and the black background was from exposure machine panel. b. The uncropped Western blot for Fig S1c comparing the Oct4 and Nanog protein level in naïve and primed hPSCs. β-tubulin (top) served as loading control. (b’) was the same gel as the image of (b) but with protein markers under bright field, and the black background was from exposure machine panel.
