# Supplementary material for: Single-cell 3D genome structure reveals distinct human pluripotent states
Source: Genome Biol. 2024 May 13;25:122. doi: 10.1186/s13059-024-03268-w (PMC11089717; doi:10.1186/s13059-024-03268-w)
Supplement: Supplementary file 4 — Additional file 4. Review history. [file 13059_2024_3268_MOESM4_ESM.docx]

Review History

**First round of review**

**Reviewer 1**

**Were you able to assess all statistics in the manuscript, including the appropriateness of statistical tests used?**

Yes, and I have assessed the statistics in my report.

**Comments to author:**

In this manuscript, the authors compared the 3D chromatin architecture of primed hESC and naïve hESC using the recently developed single-cell Dip-C method. They found that naïve hESC exhibits relaxed chromatin structures in the nuclear center, where naïve pluripotent genes are enriched, while primed hESC shows condensed chromatin in the nuclear center and loose chromatin in the nuclear periphery, where primed pluripotent genes localize. Combined with RNA-seq, ATAC-seq, CTCF, H3K9me3, and H3K27me3 CUT&Tag data, the Dip-C analysis revealed distinct 3D chromatin architecture, chromatin states, and transcription regulation in the two pluripotent states of hESCs. Overall, the paper is very interesting as the authors employed a state-of-the-art single-cell technology to revisit a long-standing question. However, there are some additional concerns that need to be addressed before the manuscript is suitable for publication in Genome Biology.

Major points:

1. Fig1 and Fig2: quantitative and statistical analysis is required in these figures to draw conclusions such as "more dispersed distribution" and "higher chromatin intermingling".

2. Fig 2B: please highlight the regions that show higher chromatin intermingling in prime hESCs as this conclusion is not evident in this figure. Besides, quantitative values such as chromatin intermingling index need to be calculated with proper statistics to better compare the 3D chromatin architectures of prime and naïve hESCs.

3. Only five single cells were investigated for each state. Given that single-cell 3D genomes are intrinsically stochastic and dynamic, how did the authors determine the minimal number of cells required to return statistically similar contacts?

4. Most conclusions in this study (e.g., chromatin intermingling, out-inside and inside-out chromatin distribution) do not appear to reply on single-cell analysis. The authors are suggested to generate bulk-cell Hi-C data or re-analyzed published bulk-cell Hi-C data of naïve and primed hESCs to confirm some of their findings.

5. The authors often suggest a causal relationship from the data that does not allow to draw such conclusions. Examples in the abstract include:

a. … we uncover that remodeling genome structure governs pluripotent state of human ESCs…

b. … naïve pluripotent state is achieved by remodeled active euchromatin compartment…

c. … this unique genome organization facilitates elevated chromatin accessibility on enhancers …

d. … primed state is created by intermingling genome organization…

e. … inversion or relocation of heterochromatin to euchromatin compartmentation regulates chromatin accessibility…

6. Haploid imputation is a critical computational step in this study. Please provide information regarding how a list of phased SNPs used in this study is generated.

7. Page 20 Line 16: displaying 3D chromatin structure of the two diploid chromosomes of pluripotent stem cells had already been achieved in hybrid mESCs in Tan et al., 2018, using data downloaded from Nagano et al., 2017 and the Dip-C computational pipeline.

8. Page 22 Line 33: was the "frequency of CpG" calculated from the bin itself or the contacting bins? This need to be clarified here and in the following text to avoid confusion. Please also provide sufficient details in the method part.

9. Fig 3: the ATAC-seq and H3K9me3, H3K27me3 CUT&Tag data are suggested to be assigned to maternal and paternal alleles as well to better support the conclusion that maternal and paternal X chromosomes are different in both chromatin structure and chromatin states.

10. Fig 4: the compartment A/B defined in Lieberman-Aiden et al., 2009 is suggested to be mapped to the 3D chromatin architecture to compare with the CpG frequency map.

11. Fig 5C: Are the 8 cells naïve or primed hESCs?

12. Fig 5D-E: Please proved sufficient details regarding how these figures were plotted in the corresponding figure legend. Since "CpG frequency" was used as a proxy for euchromatin, what does "CpG frequency" for naïve genes mean and how was it calculated? It is also not clear why "CpG frequency" for naïve genes decreased with increasing distance to the nuclear center in both naïve and primed cells (Page 29 Line 36-52).

13. Fig 6A: Please include naïve genes in primed cells and primed genes in naïve cells as the control.

14. Page 33 Line 19-22: This sentence is confusing, please revise it.

Minor points:

1. Some axes in the figures lack labels (e.g., Fig 4F, Fig 5A-B, 6B).

2. Please provide a supplementary table summarizing the sequencing information of the Dip-C data of the 10 single-cells, as well as ATAC-seq and CUT&Tag data of bulk cells.

3. Which cells are shown in Fig 2C, and why are the two individual cells chosen? Please demonstrate they are indeed representative. This question also applies to Fig 4D-E and 6C.

4. Page 7 Line 21: The authors mentioned that META tags help reduced loss of DNA contacts in bulk Hi-C assays, but did not explain how they improve the efficiency in single-cell Dip-C.

5. Page 8 Line 19: In addition to the github link, Tan et al., 2018 should also be cited here.

6. Page 19 Line 41: Fig 2 a, b needs to be capitalized.

**Reviewer 2**

**Were you able to assess all statistics in the manuscript, including the appropriateness of statistical tests used?**

Yes, and I have assessed the statistics in my report.

**Comments to author:**

This manuscript reports single-cell 3D genome structure analyses in naïve and primed states of H9, a human embryonic stem cell derived cell line (hESC). The authors profiled 8 cells at each state by Dip-C. This revealed distinct spatial organizations between the two pluripotent states. The naïve state has active euchromatin in the nuclear center and repressed heterochromatin at the nuclear periphery while the primed state has the opposite pattern. These compartment organizations are correlated with the differential chromatin and expression profiles of naïve and primed pluripotent genes examined by bulk omics (RNA-seq, ATAC-seq and CUT&Tag) and imaging. Based on these findings, the authors propose that reorganization of heterochromatin to euchromatin in the nuclear center regulates the conversion of the primed state to the naïve state in hESCs. This novel finding differs from typical active/repressive compartment changes often occurring at the nuclear periphery in stem cell differentiation or reprograming or in mouse primed and naïve ESCs. The analyses presented are convincing and provide new insights in the role of 3D genome organization in regulating pluripotent states of hESCs, though mechanistic investigations are lacking in this study. A few points need to be clarified to strengthen this manuscript.

1. This study is exclusively based on the H9 hESC line, which was one of the early hESC lines originally derived in the later primed state. While H9 with non-random XCI can be toggled in reverse to naïve, the naïve state could be different from that in naïve hESC lines directly derived from early embryos, e.g., Elf1 (Ware et al., 2014). This limitation should be discussed.

2. Given that haplotype information could be derived from the Hi-C data in this study or from other published Hi-C studies done in H9 (e.g., the bulk Hi-C analysis from Bin Ren's group), it would be useful to derive allelic profiles of gene expression and chromatin configuration for the whole maternal and paternal X chromosomes and selected X-linked regions shown in Figure 3 or in a supplementary figure. In addition, the authors should show the gene track in Fig. 3A, C. While CTCF is often decreased on the X when it becomes inactivated during differentiation, there is a dramatic increase of CTCF on the X in the primed state in which the X is inactivated versus the naïve state in which the X is not. Allelic profiles will show if this occurs on the paternal inactive X or the maternal active X.

3. In Fig 5C, the location of primed genes in both states should be shown as well as that of naïve genes in both states. The genes used for the analysis should be listed. This would clearer if including the chromatin distribution as shown in Fig. 4A as overlay.

4. Fig 5D and its description are confusing. Again a comparison with Fig. 4B. would help. This is an important figure for the argument made by the authors that the genes do not change position.

5. Supplementary figures showing genome-wide changes of chromatin accessibility, CTCF binding and histone modifications should be provided.

6. There are mistakes including missing or wrong labels/descriptions in figure panels and legends:

- Fig 1C appears to be a heatmap clustering of gene expression probably from RNA-seq while the legend mentions Q-PCR shown in supp Fig 1B. OCT4 should be labeled as POU5F1. GESA analysis in Fig 1D and supp Fig 1F is not described in the Method.

- Fig 3B seems to show the X structure for four single cells in each state while the legend mentioned a single cell. Xist should be XIST in Fig 3E.

- In Fig 4B, the Y-axis is confusing. The same scale should be used for each state. In Fig 4D, there is no CpG frequency shown and some of the labels of the y-axes are missing. No color key is shown for Fig 4E and the difference of primed vs. naïve is hard to see in this panel as well as in supp Fig 3E.

- Some panels have X/Y marked while there is no Y in H9, e.g., Fig 5C.

- The legend of Fig 6B should mention that this is ATAC-seq signal and that two replicates are shown for each state.

- The legend of supp Fig 4B should define the values of rows and columns. No gene track is shown in supp Fig 4C.

**Authors Response**

**Point-by-point responses to the reviewers’ comments:**

Reviewer #1:

In this manuscript, the authors compared the 3D chromatin architecture of primed hESC and naïve hESC using the recently developed single-cell Dip-C method. They found that naïve hESC exhibits relaxed chromatin structures in the nuclear center, where naïve pluripotent genes are enriched, while primed hESC shows condensed chromatin in the nuclear center and loose chromatin in the nuclear periphery, where primed pluripotent genes localize. Combined with RNA-seq, ATAC-seq, CTCF, H3K9me3, and H3K27me3 CUT&Tag data, the Dip-C analysis revealed distinct 3D chromatin architecture, chromatin states, and transcription regulation in the two pluripotent states of hESCs. Overall, the paper is very interesting as the authors employed a state-of-the-art single-cell technology to revisit a long-standing question. However, there are some additional concerns that need to be addressed before the manuscript is suitable for publication in Genome Biology.

Major points:

1.Fig1 and Fig2: quantitative and statistical analysis is required in these figures to draw conclusions such as "more dispersed distribution" and "higher chromatin intermingling".

*Response: Thank you very much for your suggestions. We have conducted data statistics and graphical presentation on the differences in chromatin intermingling between primed and naive cells in Figure 2e-f.*

2.Fig 2B: please highlight the regions that show higher chromatin intermingling in prime hESCs as this conclusion is not evident in this figure. Besides, quantitative values such as chromatin intermingling index need to be calculated with proper statistics to better compare the 3D chromatin architectures of prime and naive hESCs.

*Response: Thank you very much for your suggestions, the region with a high degree of chromatin mixing is chromosome 19. We presented the distribution of male and female parents of chromosome 19 in Figure 2g, and compared the difference in the number of contacts between Primed cells and Naive cells (Figure 2f). We counted the overall contacts of the cells and conducted cluster analysis. Primed cells and Naive cells showed a significant clustering trend (Figure 2d). And the contact 3D maps (Figure 2h) of Primed cells and Naive cells were displayed, showing a more pronounced intermingle trend in Primed cells.*

3.Only five single cells were investigated for each state. Given that single-cell 3D genomes are intrinsically stochastic and dynamic, how did the authors determine the minimal number of cells required to return statistically similar contacts?

*Response: Thank you very much for your suggestions, we made 12 samples for each type of cell (including naive and primed), but the other sequencing data had a smaller amount of about 10g. In the article, it was mentioned that not all cell chromatins exhibit the distribution pattern shown in the figure, and the ratio of the standard pattern is approximately 70%.*

4.Most conclusions in this study (e.g., chromatin intermingling, out- inside and inside- out chromatin distribution) do not appear to Response on single-cell analysis. The authors are suggested to generate bulk-cell Hi-C data of naïve and primed hESCs to confirm some of their findings.

*Response: Thank you very much for your suggestions, we reanalyzed the published bulk cell HiC data of ESCs and H9, and analyzed the overall CpG frequency distribution (Fig 4f).*

5. The authors often suggest a causal relationship from the data that does not allow to draw such conclusions. Examples in the abstract include:

a. … we uncover that remodeling genome structure governs pluripotent state of human ESCs…

b. … naïve pluripotent state is achieved by remodeled active euchromatin compartment…

c. … this unique genome organization facilitates elevated chromatin accessibility on enhancers …

d. … primed state is created by intermingling genome organization…

e. … inversion or relocation of heterochromatin to euchromatin compartmentation regulates chromatin accessibility…

*Response: We totally understand the reviewer’s concern. To ensure the accuracy of the conclusions, we modified these sentences: a.”we uncover that remodeling genome structure governs pluripotent state of human ESCs” is changed to “we uncover that remodeling genome structure is highly associated with pluripotent state of human ESCs”; b. “naïve pluripotent state is achieved by remodeled active euchromatin compartment” is slightly modified to “naïve pluripotent state may be achieved by remodeled active euchromatin compartment”; c.” this unique genome organization facilitates elevated chromatin accessibility on enhancers” is changed to “this unique genome organization is correlated with elevated chromatin accessibility on enhancers”; d.” primed state is created by intermingling genome organization” is changed to “primed state shows intermingled genome organization”; e.” inversion or relocation of heterochromatin to euchromatin compartmentation regulates chromatin accessibility” is modified to “inversion or relocation of heterochromatin to euchromatin compartmentation is related to regulating chromatin accessibility”. Thanks again for your valuable suggestion.*

6.Haploid imputation is a critical computational step in this study. Please provide information regarding how a list of phased SNPs used in this study is generated.

*Response: Thank you very much for your suggestions, an example SNP file for the GM12878 cell line (in hg19 coordinates), based on the 2016-1.0 release of Illumina Platinum Genomes. We have provided the SNP file in the Supplementaryary document 10.*

7. Page 20 Line 16: displaying 3D chromatin structure of the two diploid chromosomes of pluripotent stem cells had already been achieved in hybrid mESCs in Tan et al., 2018, using data downloaded from Nagano et al., 2017 and the Dip-C computational pipeline.

*Response: Thank you very much for your suggestions. Tan et al. used Dip-C technology to analyze the chromatin structure of mouse embryonic stem cells. This manuscript is the first to utilize Dip-C technology to analyze the diploid chromatin structure of human embryonic stem cells.*

8. Page 22 Line 33: was the "frequency of CpG" calculated from the bin itself or the contacting bins? This need to be clarified here and in the following text to avoid confusion. Please also provide sufficient details in the method part.

*Response: Thank you very much for your suggestions, the "frequency of CpG" calculated from the bin itself. We haved added Supplementaryary CpG frequency algorithm in Methods.*

9. Fig 3: the ATAC-seq and H3K9me3, H3K27me3 CUT&Tag data are suggested to be assigned to maternal and paternal alleles as well to better support the conclusion that maternal and paternal X chromosomes are different in both chromatin structure and chromatin states.

*Response: Thank you very much for your suggestions, the ATAC-seq and CUT&Tag data cannot distinguish between maternal and paternal.*

10. Fig 4: the compartment A/B defined in Lieberman-Aiden et al., 2009 is suggested to be mapped to the 3D chromatin architecture to compare with the CpG frequency map.

*Response: Thank you very much for your suggestions, we reconstructed the A/B part figure according to the department A/B defined in Lieberman Aiden et al., 2009, which showed a certain difference from the previous use of CpG frequency. Of course, there is also a similar trend of Primed central aggregation of more B parts (Fig 4e).*

11. Fig 5C: Are the 8 cells naïve or primed hESCs?

*Response：Thank you so much for your careful check. We have updated Fig 5C and added the distribution of naive genes and primed genes in both pluripotent states. The top are five primed hESCs and the bottom are five naive hESCs.*

12. Fig 5D-E: Please proved sufficient details regarding how these figures were plotted in the corresponding figure legend. Since "CpG frequency" was used as a proxy for euchromatin, what does "CpG frequency" for naïve genes mean and how was it calculated? It is also not clear why "CpG frequency" for naïve genes decreased with increasing distance to the nuclear center in both naïve and primed cells (Page 29 Line 36-52).

*Response: Thank you very much for your suggestions. the higher the CpG frequency, the higher the accessibility of chromatin. CpG frequency is based on Dip-C（ https://github.com/tanlongzhi/dip-c ）calculated by the gpc.sh script.*

13. Fig 6A: Please include naïve genes in primed cells and primed genes in naïve cells as the control.

*Response: Thank you for your rigorous consideration. In the revised manuscript, the updated Fig 6A include naive genes in primed cells and primed genes in naïve cells as the control (Supplementary 8).*

14. Page 33 Line 19-22: This sentence is confusing, please revise it.

*Response: Thank you so much for your careful check. We feel sorry for our carelessness.*

Minor points:

1. Some axes in the figures lack labels (e.g., Fig 4F, Fig 5A-B, 6B).

*Response: We gratefully appreciate for your valuable suggestion. we performed principal component analysis (PCA) on the compartment vectors of all cells (Fig 4F, Fig 5A-B,), and we have added the lables with Y axe “PC 2” and X axe “PC 1” on the Fig 4F, Fig 5A-B. The Y axe of Fig 6B shows the ATAC peak values, to which we have added the detailed values. We feel sorry for our carelessness.*

2. Please provide a Supplementaryary table summarizing the sequencing information of the Dip-C data of the 10 single-cells, as well as ATAC-seq and CUT&Tag data of bulk cells.

*Response: Thank you very much for your suggestions. We provide a Supplementaryary table summarizing the sequencing information of the Dip-C data of the 10 single-cells, as well as ATAC-seq and CUT&Tag data of bulk cells (Supplementary 9).*

3. Which cells are shown in Fig 2C, and why are the two individual cells chosen? Please demonstrate they are indeed representative. This question also applies to Fig 4D-E and 6C.

*Response: Thank you very much for your suggestions. We calculated the intermingle ratios of Chr1-ChrX in both naive and primed cells (Fig 5d). The intermingle of individual chromosomes in naive cells and primed cells has a relatively unified trend. All cells shown in figure are randomly selected.*

4. Page 7 Line 21: The authors mentioned that META tags help reduced loss of DNA contacts in bulk Hi-C assays, but did not explain how they improve the efficiency in single-cell Dip-C.

*Response: Thank you for your rigorous consideration. Methods for chromatin 3D structure capture including 3C, Hi-C and Dip-C, apply the same basic principles of Tn5 transposition. To accomplish DNA interruption, the P5 and P7 terminal partial adapters (Adapter 1/2) and the transposon end sequences are used to form a coating adapter to further assemble a Tn5 transposable complex with Tnp. This complex interrupts the receptor DNA and forms DNA with a P5 partial adapter (Adapter 1) at one end and a P7 partial adapter (Adapter 2) at the other end. Then, the barcodes are added by PCR to form a sequencing library. In another approach, the barcode and the transposon end sequence form a coating adapter and further form a Tn5 transposon complex with Tnp. Once transposition is complete, the adapter is added by PCR. However, in the actual operation process, when we use the Nextera kit (Illumina) to construct sequencing libraries, two diﬀerent barcodes are randomly inserted into the incoming DNA. These two barcodes then act as two PCR primers to amplify the resulting fragments. Reads with two diﬀerent barcodes at both ends of each read are enlarged, while reads with two identical tags are lost. As a result, at least 50% of the input DNA is lost after PCR ampliﬁcation. In multiplex end-tagging ampliﬁcation (META), the loss of input DNA is markedly reduced by inserting 20 diﬀerent barcodes. Thus, only 1/20 of the initial DNA is lost.*

5. Page 8 Line 19: In addition to the github link, Tan et al., 2018 should also be cited here.

*Response: Thank you for your question, we have added the citations to this article.*

6. Page 19 Line 41: Fig 2 a, b needs to be capitalized.

*Response: Thank you for your patience, we have changed Fig2a, b to Fig2A, B.*

**Reviewer** **#2**:

This manuscript reports single-cell 3D genome structure analyses in naïve and primed states of H9, a human embryonic stem cell derived cell line (hESC). The authors profiled 8 cells at each state by Dip-C. This revealed distinct spatial organizations between the two pluripotent states. The naïve state has active euchromatin in the nuclear center and repressed heterochromatin at the nuclear periphery while the primed state has the opposite pattern. These compartment organizations are correlated with the differential chromatin and expression profiles of naïve and primed pluripotent genes examined by bulk omics (RNA-seq, ATAC-seq and CUT&Tag) and imaging. Based on these findings, the authors propose that reorganization of heterochromatin to euchromatin in the nuclear center regulates the conversion of the primed state to the naïve state in hESCs. This novel finding differs from typical active/repressive compartment changes often occurring at the nuclear periphery in stem cell differentiation or reprograming or in mouse primed and naïve ESCs. The analyses presented are convincing and provide new insights in the role of 3D genome organization in regulating pluripotent states of hESCs, though mechanistic investigations are lacking in this study. A few points need to be clarified to strengthen this manuscript.

1. This study is exclusively based on the H9 hESC line, which was one of the early hESC lines originally derived in the later primed state. While H9 with non-random XCI can be toggled in reverse to naïve, the naïve state could be different from that in naïve hESC lines directly derived from early embryos, e.g., Elf1 (Ware et al., 2014). This limitation should be discussed.

*Response: We gratefully appreciate for your valuable suggestion. We discuss the differences and limitation in the Discussing part. “Actually, there are some differences between that in naïve hESC lines directly derived from early embryo and that from this article. The naïve cells we used in this article is derived from the conversion of hESCs line H9 by LTR7-GFP reporter, and maintained HERVH	hyper-activation which is required for human pluripotency. The expression profile of naïve cells enriched using the HERVH reporter in this article was closest resembling	the	inner cell mass when compared with the naïve cells obtained in Ohad Gafni et al. (2013, Nature. doi: 10.1038/nature12745) and showed a similar XIST expression, H3K27me3 distribution pattern around X chromosome (Jichang Wang et al, 2014, Nature), however, as well as the the naive state in this article (Jichang Wang et al, 2014, Nature) are not further assessed by functionality performing human-mice chimeras and epigenetic signature including DNA methylation and prominent deposition of H3K27me3“*

2. Given that haplotype information could be derived from the Hi-C data in this study or from other published Hi-C studies done in H9 (e.g., the bulk Hi-C analysis from Bin Ren's group), it would be useful to derive allelic profiles of gene expression and chromatin configuration for the whole maternal and paternal X chromosomes and selected X-linked regions shown in Figure 3 or in a Supplementaryary figure. In addition, the authors should show the gene track in Fig. 3A, C. While CTCF is often decreased on the X when it becomes inactivated during differentiation, there is a dramatic increase of CTCF on the X in the primed state in which the X is inactivated versus the naïve state in which the X is not. Allelic profiles will show if this occurs on the paternal inactive X or the maternal active X.

*Response: Thank you very much for your suggestions. At present, the sequencing data of bulk cells, including ATAC-seq, RNA-seq, Cut-tag, cannot accurately distinguish whether the chromatin comes from the paternal or maternal parent. Only single or several SNP in the fragments obtained by ATAC-seq or Cut&Tag-seq to distinguish parental alleles result in a high false positive rate, So we can only capture sequencing signals from an overall perspective and do not show the allelic profiles of CTCF binding on the paternal or maternal X. And we updated Fig. 3A, C with the gene track.*

3. In Fig 5C, the location of primed genes in both states should be shown as well as that of naïve genes in both states. The genes used for the analysis should be listed. This would clearer if including the chromatin distribution as shown in Fig. 4A as overlay.

*Response: Thank you very much for your suggestions. We haved updated the Fig5C which shows the location of primed genes and naïve genes in both primed cells and naïve cells. And added the genes list we used as Supplementary Excels.*

4. Fig 5D and its description are confusing. Again a comparison with Fig. 4B. would help. This is an important figure for the argument made by the authors that the genes do not change position.

*Response: Thank you for your valuable suggestion，We have modified slightly the figure 5D, E and Fig 4B in order to understand.*

5. Supplementaryary figures showing genome-wide changes of chromatin accessibility, CTCF binding and histone modifications should be provided.

*Response: Thank you very much for your suggestions. The comments are valuable and very helpful for improving our paper, so we added relevant information on chromatin accessibility, CTCF sites, and histone modifications in the Fig3f, g.*

6. There are mistakes including missing or wrong labels/descriptions in figure panels and legends:

- Fig 1C appears to be a heatmap clustering of gene expression probably from RNA-seq while the legend mentions Q-PCR shown in supp Fig 1B. OCT4 should be labeled as POU5F1. GESA analysis in Fig 1D and supp Fig 1F is not described in the Method.

- Fig 3B seems to show the X structure for four single cells in each state while the legend mentioned a single cell. Xist should be XIST in Fig 3E.

- In Fig 4B, the Y-axis is confusing. The same scale should be used for each state. In Fig 4D, there is no CpG frequency shown and some of the labels of the y-axes are missing. No color key is shown for Fig 4E and the difference of primed vs. naïve is hard to see in this panel as well as in supp Fig 3E.

- Some panels have X/Y marked while there is no Y in H9, e.g., Fig 5C.

- The legend of Fig 6B should mention that this is ATAC-seq signal and that two replicates are shown for each state.

- The legend of supp Fig 4B should define the values of rows and columns. No gene track is shown in supp Fig 4C.

*Response: Thank you so much for your careful check.*

(1) We have corrected the legend in Fig 1C. the sentence “Quantitative real-time PCR (Q-PCR) validation of relative expression levels of marker genes for naive and primed hESCs” is replaced by” Heatmap showing relative expression of representative marker genes for naive and primed hESCs”. Also, we labeled the OCT4 as POU5F1 and added method details about GSEA analysis to the Method in the revised manuscript.

*(2)* *We corrected this word “3D structures of a single cell showing compartmentalization” to “3D structures of four single cell in naïve and primed state”. And Xist was corrected to XIST in Fig 3E.*

*(3) Thank you so much for your careful check. We have corrected the X/Y axes in Fig 4B by using the same scale. Actually, Fig 4E shows 2D heat map of each chromosome contacts and distribution of naive and primed hESCs. Because the contacts per chromosome does not vary greatly between naïve and primed cells, color differences are not noticeable in 2D heat maps.*

*(4) We have modified the Fig 5C by adding the distribution of both naïve genes and primed genes in five single primed cells(top) and five single naïve cells(bottom). The panels could be clearer.*

*(5) This legend of Fig 6B was changed from “Heat maps of signal distribution around the transcription start site (TSS) ± 3000 bp of expressed genes and average profiles of enrichment at the TSS in naive or primed hESCs” into “Heat maps of ATAC-seq signal distribution around the transcription start site (TSS) ± 3000 bp of expressed genes and average profiles of enrichment at the TSS in naive or primed hESCs. two replicates are shown for each state”.*

*(6) We have added the detailed X/Y axes. Also, the gene track was added to supply Fig 4C in the revised manuscrpit.*

*We would like to thank the referee again for taking the time to review our manuscript.*

**Second round of review**

**Reviewer 1**

The authors have addressed most of my concerns, and I only have some minor questions:

1. If a list of phased SNPs is available, it should be feasible to assign sequencing data to maternal and paternal alleles. Why can't the ATAC-seq and CUT&Tag data in this study distinguish between maternal and paternal alleles? Were they generated using a different cell line without phased SNP information?

2. Based on the authors’ response, a supplementary table summarizing all sequencing information in this study (Dip-C, ATAC-seq, and CUT&Tag) is provided as Supplement 9. However, it appears that the revised manuscript does not contain Supplement 9.

**Authors Response**

**Point-by-point responses to the reviewers’ comments:**

Reviewer #1:

The authors have addressed most of my concerns, and I only have some minor questions:

1. If a list of phased SNPs is available, it should be feasible to assign sequencing data to maternal and paternal alleles. Why can't the ATAC-seq and CUT&Tag data in this study distinguish between maternal and paternal alleles? Were they generated using a different cell line without phased SNP information?

2. Based on the authors’ response, a supplementary table summarizing all sequencing information in this study (Dip-C, ATAC-seq, and CUT&Tag) is provided as Supplement 9. However, it appears that the revised manuscript does not contain Supplement 9.

*Response: we have addressed the limitation regarding the derivation of allelic profiles of gene expression and chromatin configuration in the Discussion section. Regarding Figure 4D, as mentioned by Reviewer 2, we have corrected the legend to accurately reflect the absence of CpG frequency data. Additionally, we have clarified the axes of supplementary figure 4B in both the legend and the figure itself.*

**Third round of review**

**Reviewer 1**

I appreciate that the authors included some discussion regarding why the RNA-seq, ATAC-seq, and CUT&Tag data used in this study cannot be assigned to the maternal and paternal alleles like the Dip-C data. However, the explanation remains unclear to me. Could the authors clarify why more SNPs were found in the fragments of Dip-C data? Please provide a clearer discussion on this matter.

**Reviewer 2**

The reviewers responded in part to previous criticisms. The paper could be accepted at this point, with the caveat that it will need to be heavily edited to make it fully understandable.

**Authors Response**

**Point-by-point responses to the reviewers’ comments:**

Reviewer #1:

I appreciate that the authors included some discussion regarding why the RNA-seq, ATAC-seq, and CUT&Tag data used in this study cannot be assigned to the maternal and paternal alleles like the Dip-C data. However, the explanation remains unclear to me. Could the authors clarify why more SNPs were found in the fragments of Dip-C data? Please provide a clearer discussion on this matter.

*Response：For diploid cells, identifying parental alleles using next-generation sequencing (NGS) technologies relies primarily on the distribution of SNPs across the genome. Because SNPs are not only distributed in transcriptionally active regions but also commonly spread across non-coding regions. Distinguishing between two parental alleles of a chromosomal region using technologies based on NGS requires the presence of heterozygous single nucleotide polymorphism (SNP) sites within the region, typically ranging in length from 80 to 300 base pairs. Different techniques such as RNA-seq, ATAC-seq, when obtaining fragments and mapping them to the genome, correspond to specific regions, including exonic regions and open regions. However, due to the different sequencing principles, Dip-C can obtain fragments from all regions of the genome, thus resulting in a higher number of fragments containing SNP sites. Similarly, when using DiP-C fragments to simulate the parental chromosome states, the false positive rate would be lower. Nevertheless, it is undeniable that when examining a small fragment (80-300bp) of the genome containing SNP sites, there is no significant difference between RNA-seq, ATAC-seq, CUT&Tag, and DiP-C.*

Reviewer #2:

The reviewers responded in part to previous criticisms. The paper could be accepted at this point, with the caveat that it will need to be heavily edited to make it fully understandable.

*Response: We have submitted the manuscript to an institution for professional English native speakers to polish, correcting many grammar, vocabulary, and spelling issues to ensure improved readability.*
